# Self-optimized single-nanowire photoluminescence thermometry

**DOI:** 10.1038/s41377-023-01070-0

**Published:** 2023-02-06

**Authors:** Zhang Liang, Jinhua Wu, Ying Cui, Hao Sun, Cun-Zheng Ning

**Affiliations:** 1 Department of Electronic Engineering, Tsinghua University, 100084 Beijing, China; 2 College of Integrated Circuits and Optoelectronic Chips, Shenzhen Technology University, 518118 Shenzhen, Guangdong China

**Keywords:** Applied optics, Nanowires, Imaging and sensing

## Abstract

Nanomaterials-based photoluminescence thermometry (PLT) is a new contact-free photonic approach for temperature sensing, important for applications ranging from quantum technology to biomedical imaging and diagnostics. Even though numerous new materials have been explored, great challenges and deficiencies remain that hamper many applications. In contrast to most of the existing approaches that use large ensembles of rare-earth-doped nanomaterials with large volumes and unavoidable inhomogeneity, we demonstrate the ultimate size reduction and simplicity of PLT by using only a single erbium-chloride-silicate (ECS) nanowire. Importantly, we propose and demonstrate a novel strategy that contains a self-optimization or “smart” procedure to automatically identify the best PL intensity ratio for temperature sensing. The automated procedure is used to self-optimize key sensing metrics, such as sensitivity, precision, or resolution to achieve an all-around superior PLT including several record-setting metrics including the first sensitivity exceeding 100% K^−1^ (~138% K^−1^), the highest resolution of 0.01 K, and the largest range of sensible temperatures 4–500 K operating completely within 1500–1800 nm (an important biological window). The high-quality ECS nanowire enables the use of well-resolved Stark-sublevels to construct a series of PL intensity ratios for optimization in infrared, allowing the completely Boltzmann-based sensing at cryogenic temperature for the first time. Our single-nanowire PLT and the proposed optimization strategy overcome many existing challenges and could fundamentally impact PL nano-thermometry and related applications such as single-cell thermometry.

## Abstract (web summary)

A self-optimized single-nanowire photoluminescence thermometer allows the construction of multiple criteria for temperature measurement and chooses the best one by an automated routine based on specified optimization targets like sensitivity.

## Graphical abstract

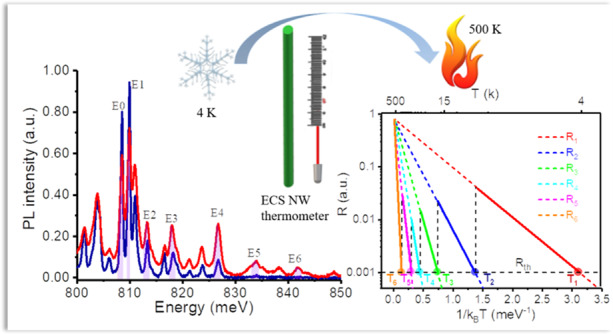

## Introduction

Accurate and sensitive determination of temperature is important for many aspects of daily life, high tech, biology, and medicine^1–6^. Traditional thermometry based on liquids expansion or electric response of metals such as resistance temperature detectors (RTD) is often unsuitable for many modern scientific and technological needs in extreme engineering environments, or in biological systems such as a single cell during cell apoptosis^7,8^. Other modern temperature sensors based on electrical signals require electrical wiring^9,10^ and are thus not suitable for many applications such as in vivo bio-medical applications^11–14^. In contrast, nano-materials-based photoluminescence thermometry (PLT), especially the photoluminescence intensity ratio (PLIR)-based approach has emerged as an important alternative with many advantages, such as immune to electromagnetic interference and contact-free measurement^15–21^. The foundation for the PLIR-based temperature measurement is the Boltzmann distribution. The PL intensity associated with a given transition is proportional to the population of the upper state, and the intensity ratio of the two transitions is a function of temperature and intrinsic parameters only, independent of the extrinsic properties. The PLIR thermometers have progressed rapidly in the past decade^21^, with new application scenarios continuously being explored^12,15,22–26^. At the same time, new materials for PLT with better thermometry characteristics are being developed constantly to meet the needs of ever-expanding application scenarios^27–34^, especially those in bio-medical^16,35^ and other areas of science and engineering^36^ where cryogenic temperature sensing is important^37–39^.

Despite a great past and continuing progress^40–42^, many significant challenges remain^16^.

The first is a materials challenge or searching for all-around high-performance PLT materials. Such high-performance materials should ideally have the best of all the key performance characteristics, such as the highest relative sensitivity and temperature resolution, highest PL efficiency, the largest range of sensible temperature in IR wavelength, and best physical and chemical stabilities^43–47^. But currently, such materials do not yet exist^9,16^.

Second, nano-materials that apply cryogenics to body temperatures are still extremely rare^48–50^. Sr_2_GeO_4_:Pr^3+^ crystalline powders were recently used to measure temperatures from 17 to 600 K^51^ using two separate sets of PLIR: one below and one above 300 K. Such multi-sets PLIR procedure requires prior knowledge of the temperature range of the to-be-measured object.

Third, for bio-medical applications, PLIR materials with both emission bands completely in the NIR-IIB (1500–1800 nm) biological window are important for deeper tissue penetration and high contrast sensing due to the suppressed light scattering and diminished tissue autofluorescence^46,52^. An interesting recent demonstration used one of the emission peaks in the NIR-IIB window to construct intensity ratios to two peaks in the NIR-I (750–900 nm) and NIR-IIA (1000–1300 nm)windows^47^, with the penetration depth still limited by these short wavelengths. Although the PLIR thermometers that operate completely in the NIR-IIB band were studied recently, they are all limited to a narrow temperature range around ambient temperatures^32,53,54^.

Fourth, the cryogenic sensor is vital for many applications such as cryobiology^37,48–51,55,56^, aerospace^57^, and solid-state superconducting quantum technologies^38^. But it is still a challenge for PLIR thermometers to work at low temperatures using Boltzmann mechanisms^31,58,59^. In the existing PLIR thermometers, typical thermally coupled levels based on the Boltzmann law are not available below 100 K due to the large energy separations involved compared to thermal energy in cryogenic hosts^60^, such as ^4^S_3/2_ and ^2^H_11/2_ levels of Er^3+^. As a result, other temperature-dependent properties (non-Boltzmannian) are used for temperature sensing^51,61^. Such non-Boltzmannian PLT suffers from difficulty and complexity of the determination of response function in the absence of Boltzmann distribution^30^, leading to complicated issues such as calibration and stability of thermometry. More importantly, thermal parameters change with other non-thermal characteristics, such as pressure or strain, leading to inaccurate temperature sensing. Stark sub-levels with small energy differences give us a unique opportunity to design the Boltzmann-based PLIR for cryogenic applications but have been rarely explored due to poor material quality. Recently Shang et al.^60^ reported one of the rare interesting cases of using the NIR Stark splitting around 801 and 820 nm from Tm^3+^ ions for the Boltzmann-type cryogenic sensor (10 K). One notices that the broad linewidth of emission leads to clear deviation from the exact Boltzmann behavior below 30 K. High-quality crystal materials are crucial for true Boltzmann-type sensing at cryogenics and it would be even more interesting to extend such sensing to the IR wavelength ranges.

The fifth issue is that the overwhelming existing PLIR approaches use an ensemble of nanomaterials such as nano-particles^12^. The large volume and/or quantity required is poorly suited for applications involving small-scale objects, such as cell-level probes where more accurate site or location control is required^20,62^. The inhomogeneity associated with an ensemble causes other issues of spectral broadening and inaccuracies^63^. These issues can be addressed by using a single nano-object for PLT, a largely unexplored subject.

To overcome key existing challenges and remedy the many deficiencies of current approaches as outlined above, we propose and demonstrate in this paper a new strategy with an automated optimization scheme based on a single-frame acquisition of the PL spectrum. The self-optimization scheme allows for the choice of the best temperature-determination criterion depending on the applications and the desired optimization targets of the users. We believe that such a self-optimization scheme can become a more generally applicable strategy for temperature sensing in various situations. Specifically, we report our results on a new PLT based on a single nanowire of erbium chloride silicate (ECS NWs)^64–66^, first developed by our group. ECS NW is a relatively new material in the rare-earth family and has a rare combination of extremely high material quality and high Er concentration. These unique properties have led to extremely high optical quality as evidenced by record-high optical gain and amplification^65^. But this material has not been explored for PLT applications. Compared to the existing PLT approaches, our ECS-based approach shows an all-around superior performance. The temperature measurement in this paper is based on the Boltzmann distribution of erbium atoms in all temperature ranges. To our knowledge, this is the first fully Boltzmann-type PLT implemented in a wide range of temperatures including cryogenic temperatures for the NIR range. The extremely high sensitivity of up to 138% K^−1^ represents the first such PLT thermometer with a sensitivity exceeding 100% K^−1^. Finally and importantly, instead of an ensemble of nanomaterials with unavoidable inhomogeneous broadening, we demonstrated the PLT sensing with a single nanowire with overall superior performance.

## Results

**Fig. 1:**
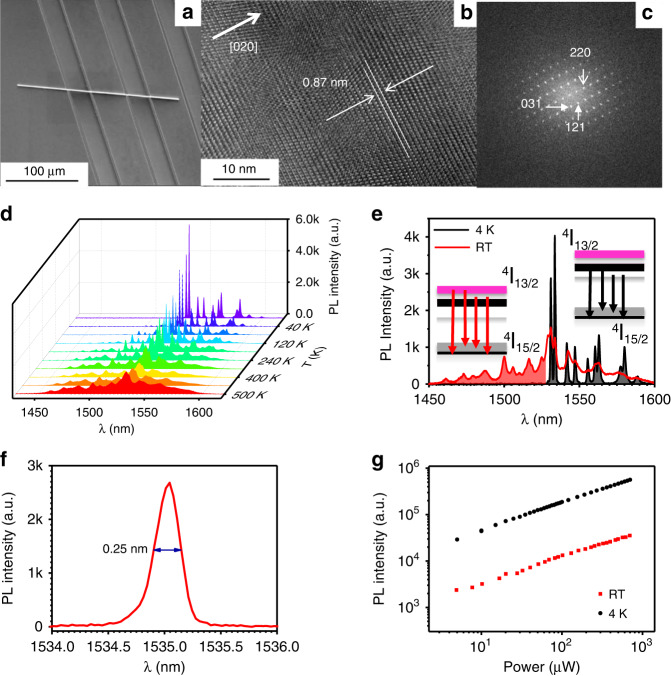
Material characterization and spectral characterization of a single ECS nanowire. **a** SEM image of a single nanowire on a substrate with parallel grooves (**a**, scale bar: 100 µm). **b** HRTEM of ECS single crystal (scale bar: 10 nm) and **c** the lattice diffraction pattern. PL properties of an ECS nanowire in the NIR-IIB bio-window pumped by 980 nm diode laser. **d** PL spectra from 4 to 500 K. **e** Comparison of PL spectra at 4 K and room temperature showing temperature-dependent population change and the corresponding PL spectral changes forming the foundation of temperature sensing mechanism. **f** Spectrum of a single Stark sub-peak originated from ^4^I_13/2_ levels at 4 K. **g** Power dependence of integrated emission intensity originated from ^4^I_13/2_ levels at 4 K and the room temperature

The conventional choice of materials for PLT is rare-earth-doped nanomaterials such as a large collection of nanoparticles with large numbers or volumes. These materials suffer from low crystal quality and non-uniform optical properties. Our material choice is Er_3_(SiO_4_)_2_Cl (ECS) which belongs to a group of compound crystals Ln_3_(SiO_4_)_2_X, where Ln represents a rare-earth element and X halogen element). The Ln_3_(SiO_4_)_2_X crystals have an orthorhombic Bravais lattice structure for heavy rare-earth compounds (Er, Y, Yb), and a monoclinic Bravais lattice structure for light rare-earth compounds (La, Ce, Pr, Nd)^67,68^. Instead of being random dopants in typical nanoparticles, Ln is periodically placed in the compounds, leading to superior material properties. Even though high-quality single-crystal materials of lanthanum chloride silicate are very difficult to produce in bulk or film forms, ECS has been produced in very high quality in nanowire form by our group^64^. We have previously studied this material extensively for its superior optical properties including the largest optical gain of any erbium materials^65,69^. The ECS nanowires are grown by the chemical vapor deposition (CVD) method and the details of material preparation are discussed in the “Methods” section. The ECS nanowires are typically perfect single crystals with high material quality, which is the physical foundation for its strong photoluminescence (PL) efficiency and narrow PL linewidth, critical for the high performance of our PLT developed here. The quality and uniformity of the as-grown ECS NW can be affirmed by the scanning electron microscope (SEM) as shown in Fig. 1a, high-resolution transmission electron microscopy (HRTEM) analysis as-shown in Fig. 1b and c, and energy-disperse X-ray spectroscopy (EDS) element analysis of line-scanning and dot detection, as shown in SI Figs. S1, S2, and Table S1 (see SI Section I). The ECS nanowires have diameters ranging from tens of nanometers up to micrometers and lengths as long as over 100 µm. The unit cell of ECS is an orthorhombic phase with *a* = 6.82 Å, *b* = 17.65 Å, and *c* = 6.16 Å. More detailed morphology and lattice information of ECS NW are given in SI Section I. The selected ECS NW was placed on a Si substrate coated with Au film. Further results of materials characterization can be found in refs. ^64,66^.

Figure 1d shows the PL spectra in the NIR-IIB window for temperatures from 4 to 500 K, originating from ^4^I_13/2_ states to ground states ^4^I_15/2_ of Er^3+^ ions in ECS nanowire, pumped by a diode laser of 980 nm. The well-separated spectral peaks shown in Fig. 1d and e (especially at low temperatures) are the results of Stark splitting due to the crystal field and are indications of the high crystal quality of our ECS nanowires. Other erbium materials typically used for light applications or PLT applications show featureless broadband emission due to poor material quality. We see that, at very low temperatures, e.g., 4 K, the PL emission has only the long-wavelength half, as electrons mostly occupy the lowest sublevels of ^4^I_13/2_ states (see also Fig. 1e). As temperature increases, the short-wavelength half starts to appear and the spectrum broadens with temperature, as higher sublevels of the ^4^I_13/2_ band are thermally occupied. Such thermally induced changes in the spectrum are the result of the thermal equilibrium distribution given by the Boltzmann distribution (thus the Boltzmannian PLT) and the physical basis for temperature sensing. Previously, most nano-erbium materials are produced with the wet chemical synthesis method in powder or crystal forms^9,12^. The materials are usually much more defective than those grown using high-temperature methods such as CVD. So, the linewidth broadening in these more defective materials is typically larger. Thanks to the excellent crystal quality, the linewidth of the PL spectrum for the Stark sublevels can be as narrow as 0.25 nm in 4 K, shown in Fig. 1f. The narrow spectral lines are very important for temperature sensing at cryogenic temperatures as in such a situation we have clearly separated Stark sub-levels following the Boltzmann distribution law. The linewidth evolution with the temperature is given in Fig. S3 in SI section I, it is clear that the linewidth in the whole sensing range is below 1 nm as measured with our detection system. The NW diameter is found to have little influence on the spectral line shape of the ECS NWs as shown in Fig. S4 in SI section I. Only the absolute value of the PL intensity is affected by the size variation of the NWs. Therefore, the PLIRs are not influenced by NW’s diameter. Figure 1g is the log–log plot of PL intensity vs. excitation power, the PL intensity follows the scaling relationship

1 $$I_{{\rm {PL}}} \propto P_{{\rm {ex}}}^n$$

where *I*_PL_ represents the measured PL intensity and *P*_ex_ is the excitation power. The power index *n* is an indication of the orders of nonlinearity. We found that both in cryogenics and room temperatures, the PL intensity depends on excitation power sublinearly, with a constant scaling power index of *n* ~ 0.5 over three orders of magnitude. The sub-linear power dependence is related to the up-conversion and other energy-transfer processes. The excitation power in our experiments was typically on the order of tens of microwatts. In summary, unlike traditional nanomaterials for PL thermometers which mostly use doping of rare-earth ions with a RE density <10^19^ cm^−3^, the ECS NW is an erbium compound with erbium density at 1.6 × 10^22^ cm^−3^. This rare combination of the high Er-density and high single crystal quality leads to the highest density–lifetime product^66^ and high PL efficiency for the 1.5 µm wavelength in the NIR-IIB band. More importantly, the high single crystal quality leads to narrow spectral line widths for the crystal-field-induced transitions, assuring the emission spectral lines around 1.5 µm are well separate to construct PLIRs.

Clearly, the PLTs require the existence of two atomic or molecular levels that are “thermally coupled” (TC)^70^, meaning the relative occupations of the two states will change appreciably with temperature in the range of temperature of interest. This golden rule is considered the foundation for PLTs. The lower limit of the sensible temperature range of a given PLT is determined by the smallest separation of well-resolved spectral peaks. Similarly, the upper limit of the sensible temperature range is determined by the largest well-resolved spectral peaks. For temperature sensing around room temperatures, the two TC levels need to have an energy gap larger than 200 cm^−1^, or 24 meV, the thermal energy *k*_B_*T* at room temperature, to avoid the PL spectrum overlapping. PLT with energy separation this large cannot be used to sense temperatures significantly below RT. Due to the lack of well-resolved spectral peaks with much smaller separation as a result of poor material quality, researchers had to resort to other non-Boltzmannian physical mechanisms that lead to temperature-dependent PL emission, to sense low temperatures. For example, light emission from material could change as a result of temperature-dependent thermal expansion of the crystal lattice of a solid. Such a mechanism could be used for sensing temperature. These mechanisms often involve linear or polynomial dependence of PLIR on temperature or even more complicated response functions^44^. While such approaches provide new opportunities for temperature sensing especially in the cryogenic range when TCLs are not available for many situations, they have certain intrinsic deficiencies. For example, the physical mechanisms of the temperature dependence of PL are often unexplained or poorly understood. The sensitivity often shows a strong dependence on molar ratios in materials and a slight variation may result in a different response to temperature. Thus material preparation and repeatability become important issues, leading to serious problems with calibration^60^. Conventional PLT starts by choosing two TCLs and constructs a PLIR such as

2 $$R = \frac{{I_2}}{{I_1}} = C \ast \exp \left( { - \frac{{E_2 - E_1}}{{k_{\rm {B}}T}}} \right)$$

where *E*_*i*_ is the energies of the state i, *k*_B_ is the Boltzmann constant. Equation (2) is the physical basis for Boltzmann-type PLT, while exponential dependence on temperature is the mathematical basis for high-sensitivity temperature sensing. Obviously, the sensible temperature range is determined by the energy separation of the two levels and the line widths of the transitions involved. The key attributes for PLT include sensible temperature range (*T*_r_), sensitivity (*S*_r_), temperature resolution (Δ*T*), precision, tissue penetration depth (for bio-medical applications and related to the emission wavelength), spectral acquisition time (*t*_A_) which determines temporal-resolution or response, and the repeatability. The physical definitions of these properties are given in detail below^16,45^. Relative sensitivity is defined as the first-order derivative of *R* with respect to temperature, divided by *R* itself

3 $$S_{\rm {r}} = \left| {\frac{{\partial R}}{{\partial T}}\frac{1}{R}} \right| = \frac{{\partial {{{\mathrm{ln}}}}(R)}}{{\partial T}} = (E_2 - E_1)/(k_{\rm {B}}T^2)$$

The relative sensitivity is proportional to the energy gap of the upper levels at the different transitions used and is inversely proportional to the square of the temperature. Therefore, at the same temperature, the greater the Δ*E*, the better the relative sensitivity. The standard deviation for intensity ratios *σ* and the signal-to-noise ratio (SNR) are also defined and given in SI Section II.

Temperature resolution is the smallest variation of temperature to induce a detectable PLIR variation and is defined as

4 $$\Delta T = \frac{\sigma }{{S_r \cdot R}} = \frac{\sigma }{{\frac{{\partial R}}{{\partial T}}}} = \frac{\sigma }{R}\frac{{kT}}{{E_2 - E_1}}T$$

which could be understood as a temperature-resolving power, which is proportional to the product of the sensitivity and SNR. Acquisition time *t*_A_ is the time needed to complete a single temperature detection and it is mainly determined by the time needed to collect a PL spectrum in a certain wavelength range with the desired SNR. For a monotonic response curve, we can deduce temperature from the corresponding PLIR value.

The choice of PLIR is very important for those key PLT attributes outlined above. But often the PLIR choice is not unique and the criteria are not often discussed. The typical approach is to choose a single intensity ratio, *R* (see Eq. (2)) and its temperature range of validity is determined by the acceptability of other attributes, defined above Eqs. (3)–(4). Typically, one selects two emission peaks with energy separation in the range between 200 and 2000 cm^−1^. Obviously, any single such selected PLIR cannot guarantee that all these attributes are optimized. To remedy these deficiencies and to greatly improve the important attributes of PLT, we propose and demonstrate a fundamentally different strategy in this paper. Our approach is enabled by the high crystal quality and the associated narrow linewidth down to 0.25 nm at low temperatures. This allows us to choose sub-levels within the Stark ladder as TCLs with an energy gap of only a few cm^−1^ (see Fig. 1f and S7), allowing Boltzmannian sensing for much lower temperatures down to a few K for the first time.

**Fig. 2:**
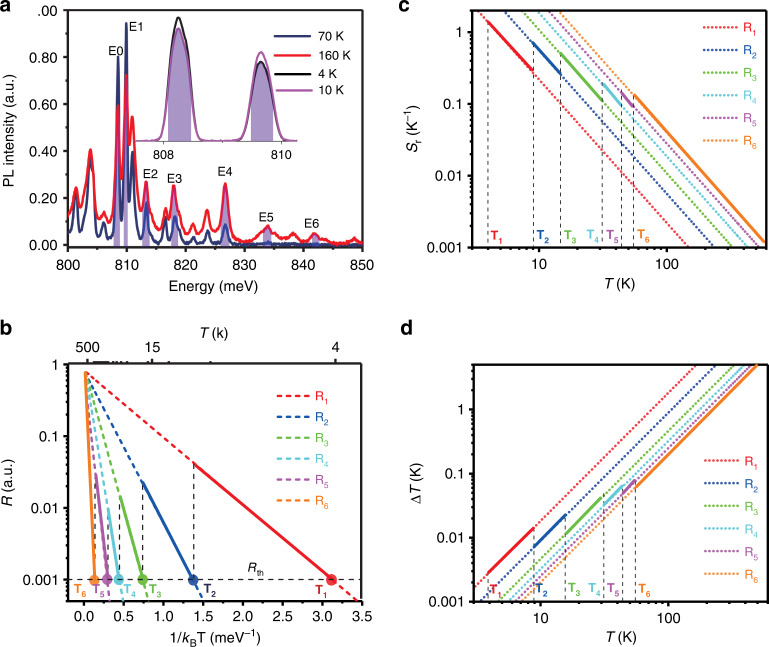
Construction of intensity ratios and measurement strategy. **a** Spectra between 800 meV (1.55 μm) and 850 meV (1.46 μm) showing key spectral peaks selected to construct PLIRs; **b**) Temperature dependence of various PLIRs, *R*_1_ through *R*_6_, with both temperature (top axis) and inverse temperature scaled by the Boltzmann constant (low axis). The segment with solid lines indicates the range of temperature where a particular *R*_*i*_ is used, because of larger sensitivity (see **c**). **c** Sensitivities and **d** temperature resolution for the six constructed PLIRs, *R*_1_–*R*_6_, respectively. The segments with solid lines indicate the sensitivities and the temperature resolutions corresponding to *R*_*i*_’s used for sensing consistent with color code and convention in (**b**)

Due to the strong PL emission of ECS nanowires, one can rapidly collect an emission spectrum in a relatively wide range, such as those presented in Figs. 1d, e, and 2a. Such a spectrum contains often more spectral features (such as peaks), representing several strong emission channels. Instead of constructing a single PLIR, we can construct a series of PLIRs using these peaks. There are two ways to construct these PLIRs. One is to use measured spectra such as those in Figs. 1d, e, and 2a. Alternatively, we can also construct such PLIRs more generally based on known energy levels and transitions, as is presented in SI Section III. The 980 nm pump laser populates ^4^I_11/2_ states directly. The rapid thermalization leads to a dynamic equilibrium, the Boltzmann distribution among the ^4^I_13/2_ Stark sublevels. The transitions between ^4^I_13/2_ and ^4^I_15/2_ sub-levels generate multiple sub-peaks around 1530 nm, reflecting the dynamic equilibrium Boltzmann distribution among the Stark sub-levels. This provides the physics foundation for the Stark sublevels-based PLIR thermometry. At the same time, the multi-peak feature provides an opportunity to construct multiple PLIRs, allowing us to choose the best one for thermal sensing based on optimization, which can be further automated as described later on (see SI Sections III and VIII). In the following, we outline our strategy of a more general and systematic PLT using the spectrum that corresponds to the ^4^I_13/2_ to ^4^I_15/2_ transitions as follows:

1. Determine the reference peak, called *E*_0_, which corresponds to emission transitions from the lowest excited states, say from ^4^I_13/2_ states to ^4^I_15/2_ states. This peak is the strongest one at the lowest temperature such as *E*_0_ at 4 K in Fig. 1d and e. In general, the excited states need to be populated at the lower boundary of the sensible temperature range.
2. Start from *E*_0_ and select a series of strong emission peaks, *E*_1_, *E*_2_,…, from the measured spectrum with more or less similar separations such that *E*_0_ < *E*_1_ < … < *E*_6_…, as marked in Fig. 2a. The integrated intensities of these peaks are denoted as *I*_*m*_ (*m* = 0, 1, 2,…). These peaks correspond to emissions from successively higher states with an increase in temperature.
3. Construct PLIRs relative to the reference peak (*E*_0_): *R*_*m*_ = *I*_*m*_ /*I*_0_ (*m* = 1, 2,*…*). In our construction of the PLIRs, the areal integration of each peak within the full width at half maximum (FWHM) is used to represent the total intensity of each peak, as carried out by MATLAB and discussed in the previous version SI section VIII.
4. Determine the correct PLIRs that can be used as a measurement of temperature. From Eq. (2), it follows that

5 $$\ln \left( {R_m} \right) = \ln \left( C \right) + \left( {E_m - E_0} \right)\frac{1}{{k_{\rm {B}}T}}$$

If we plot ln(*R*_*m*_) vs. 1/(*k*_B_*T*) as shown in Fig. S7 of SI Section IV (experimental data with fitted line), the slope will be *E*_*m*_−*E*_0_ for the Boltzmann distribution. The slope for each of these plots, *S*_*m*_, is compared to Δ*E*_*m*_ = *E*_*m*_−*E*_0_. Only those *R*_*m*_ for which *S*_*m*_ = Δ*E*_*m*_ within the margin of the linewidth of corresponding transitions can be used as a PLIR for temperature measurement. These *R*_*m*_’s are plotted in Fig. 2b. As shown in Fig. S8 of SI Section V, not all emission peaks in the measured spectrum correspond to emissions from different excited states. We only need those emission peaks that follow the Boltzmann distribution. This process is further explained in SI Sections IV and V.

We now have a series of *R*_*m*_’s that can be used for temperature determination as shown in Fig. 2b. To have a large enough signal-to-noise ratio (SNR) of our measurement (see Eq. (S3)), we require that these *R*_*m*_’s to be larger than a certain threshold, *R*_th_, for which SNR = 20 dB (a feasible value readily achieved in our experiments, see SI Section VII for details). The intersections of *R*_th_ with *R*_*m*_’s define a series of *T*_*m*_’s for each of *R*_*m*_’s, as shown in Fig. 2b. These *T*_*m*_’s determine the lower limit of the temperature sensing range of *R*_*m*_’s. *T*_1_ sets the overall lower limit of the temperature range that can be sensed by our PLIR thermometer.

The availability of this series of *R*_*m*_’s gives us great flexibility in choosing which of the *R*_*m*_’s to be used (for what temperature range) for temperature sensing by a fully automated optimization process. For a given application, the optimization process can be accomplished in a fully automated manner depending on the need of a given application by having one or a few of the attributes optimized such as maximized sensitivity, or highest temperature precision, etc. Here we illustrate our approach using the maximization of sensitivity as an example. To begin with the measurement process, we first use *R*_*1*_ to measure temperature, since *R*_1_ covers the largest temperature range with *R*_1_ > *R*_th_ (see Fig. 2b) from 4 to 500 K. In principle, *R*_1_ can be used for temperature sensing in the entire 4–500 K range. Similarly, *R*_2_ can be used for temperatures between *T*_2_ and 500 K. But from Fig. 2c, we see for each of the *R*_*i*_, the sensitivity decreases as temperature increases. Therefore, for *T* > *T*_2_, *R*_2_ has higher sensitivity (Fig. 2c) and *R*_2_ should be used. Similarly, for *T* > *T*_3_, *R*_3_ should be used, etc. This process is continued so that for any to-be-measured temperature between 4 and 500 K, we always find the right *R*_*i*_ with the highest sensitivity. In our example of having 6 PLIRs, *R*_6_ is our last choice for temperature sensing. Using this procedure, the final choices of *R*_*i*_’s are indicated by the segments with solid lines in Fig. 2b. This simple routine is automated with a computer code given in the SI Section VIII. The automatic program will estimate the temperature with *R*_1_ depending on the measured spectrum and then find the suitable response curve with the best concerning parameters, such as the maximum sensitivity. In application, the only thing we need to do is to make the single frame spectrum acquisition and run the program in SI Section VIII. The program will finish the measurement with the maximum sensitivity response curve in *R*_1_–*R*_6_. Interestingly, our procedure described above for maximization of sensitivity also is simultaneously optimized to have the highest temperature resolution. The theoretical values of the temperature resolution are also given in Fig. 2d (segments with solid lines). The five steps outlined above represent our systematic procedure to an automated temperature sensing strategy and the procedure is generally applicable to a wide range of PL materials and situations. We will demonstrate this procedure using the example of an ECS nanowire, especially the well-resolved Stark peaks due to high crystal quality (see Fig. 1e and f). Thermally coupled Stark sublevels were mostly used for PLT at room temperature in UV–VIS wavelengths or short-wavelength IR ranges (~800 nm). It is clear, however, that the small energy differences between Stark sublevels are ideally suited for Boltzmann-type sensing at cryogenic temperatures. But previous such attempts are obscured by the low quality of materials that resulted in broad linewidths. Due to the unavoidable finite linewidth (*W* > 0), the measurement based on the Stark sublevels is meaningful when the energy separation of the Stark sublevels, Δ*E*, is larger than *W*. The temperature resolution Δ*T* is determined by *σ*/(*R*·*S*_r_). A wide linewidth that is bigger than Δ*E* would induce big *σ* for *R*, and cause fluctuations of Δ*E* which in turn lead to the fluctuations of *S*_r_ (Δ*E*/*k*_B_*T*^*2*^). The big *σ* and the fluctuations of *S*_r_ would lead to a poor temperature resolution. This is actually the key advantage of our high-quality single-crystal material that has the narrowest linewidth among high Erbium materials. The combination of our high-quality crystal material with sharp spectral lines (see Fig. 2a) and the optimization procedure that allows us to select the best sensing PLIR will lead to highly sensitive PLT performance. This can be easily seen by comparing the self-optimized sensing with the classical (traditional) approach which corresponds to using one of the six sensing criteria for the entire temperature range. In the traditional PLIR method, suppose we use the *R*_3_ as the temperature criterion for the entire temperature region. First, all temperatures below the *T*_3_ cannot be sensed, limiting the range of sensing temperature significantly as shown in Fig.2c. Second, for temperatures above *T*_4_, sensing using the traditional approach by *R*_3_ would have much lower sensitivity than using the self-optimized one. The same is true for the temperature resolution from Fig. 2d.

**Fig. 3:**
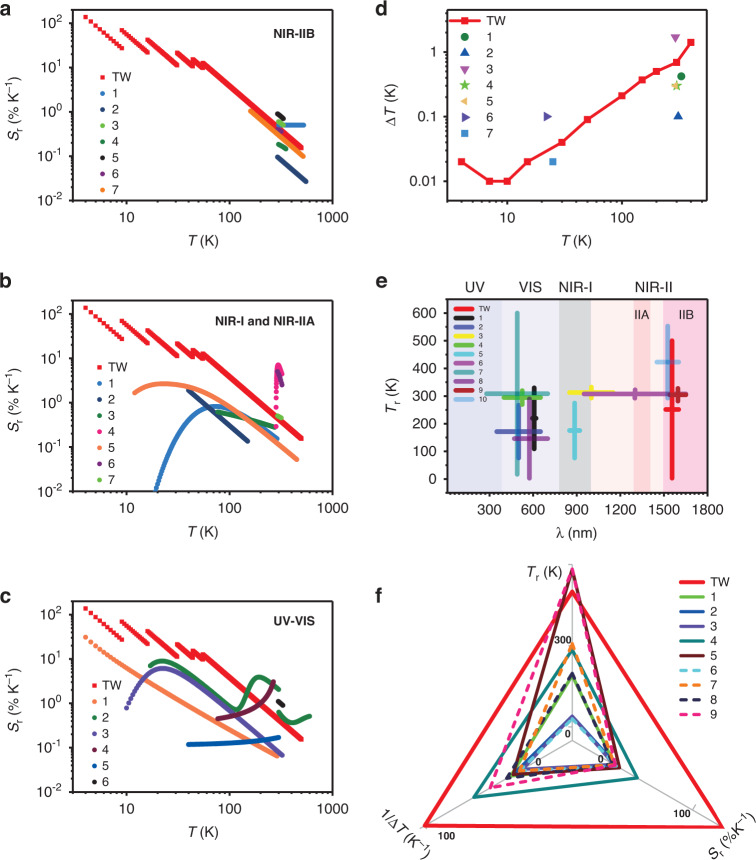
Comparisons of key performance characteristics of this paper with Existing Approaches (TW: this work). **a**–**c** Comparison of relative sensitivities of our approach with available best data in literature working in the pure NIR-IIB, UV–VIS, and NIR-I/NIR-IIA band, respectively. **a** 1–7 represent refs. ^54,71,75–79^, respectively. **b** 1–7 represents refs. ^12,44,47,58,60,80,81^, respectively. **c** 1–6 represents refs. ^25,30–33,51^, respectively. **d** Comparison of temperature resolutions with literature (1–7 represent refs. ^5,12,28,31,44,47,51^, respectively). **e** Comparison of sensible temperature ranges vs. wavelengths (1–10 represent refs. ^5,28,30,33,43,47,51,54,58,76^, respectively), where the length of the vertical bars represent the temperature ranges of various sensing approaches while the width of the horizontal bars represents the range of wavelengths used. **f** A summary comparison of key characteristics with literature (1–9 represent refs. ^12,30,33,47,51,56,58,71,72^, respectively)

Figure 3 shows the key performance attributes of our PLT based on the optimized sensitivity, compared with performance parameters in the literature. In Fig. 3a–c, we compare our sensitivities with those from literature with high sensitivity covering working wavelengths in the NIR-IIB, NIR-I&NIR-IIA, and UV–VIS bands. We can see from Fig. 3a that in the NIR-IIB band, our approach covers the largest temperature range with the highest cryogenic sensitivity. Although the thermometer in ref. ^71^ can reach 0.9% at 293 K, it is limited to a small temperature range from 293 to 333 K. Our work can still maintain competitive sensitivity around ambient temperature compared with other PLTs in NIR-IIB. Refs. ^12,47^ show significant sensitivity at room temperature listed in Fig. 3b, but parts of its emission peaks are in the NIR-IIA window. Therefore, the penetration depth is still limited by these short wavelengths. Figure 3b compares our sensitivities in NIR-IIB with those in NIR-I and NIR-IIA from the literature. We can still see clear advantages of our approach, especially its wide temperature range and high sensitivity in cryogenic temperatures. Even compared with the UV–VIS PLTs from literature (Fig. 3c), we see that the sensitivity of our PLT is larger than 100% K^−1^ at cryogenic temperatures (more specifically, 138% K^−1^ at 4 K), the largest sensitivity ever reported (which is 31% K^−1^ so far, see SI Section X). There have been very few papers on PLT sensing for temperatures below 10 K^53^, and with much lower sensitivity. For temperatures below 100 K, our approach has the largest sensitivity compared to all other approaches available. For room temperature and higher, our approach can still maintain very high sensitivity: >1% K^−1^ for *T* < 200 K and >0.1% K^−1^ for *T* < 500 K. Such performance is remarkable, especially since we use PLIRs completely in NIR-IIB wavelengths, while the other approaches are based on much shorter wavelengths. We can easily extend our approach to include visible wavelengths by including other emission bands of higher energy states, our sensitivity can be maintained at more than 1% K^−1^ in the human body temperature range with 0.1 K resolution (see SI Section IX for details).

Figure 3d shows the comparison of the temperature resolution obtained in our experiment with those of other PLTs results in the literature. ∆T is an important parameter of temperature measuring devices and is dependent on temperature. However, this important attribute is not often presented for all the temperature ranges in the literature, often only the optimal temperature resolution of the developed materials and devices is given at certain selected temperatures. We see our PLT has a much smaller temperature resolution below 100 K and reaches to the smallest resolution of 0.01 K below 10 K. Previously, the best resolution achieved was 0.02 K (see also, SI Section X Table S3)). Temperature resolution increases to 0.69 K at 300 K, and finally to 3.58 K at 500 K. As we said above, if we combine the ^2^H_11/2_ and ^4^S_3/2_ traditional TC levels in the visible band, we can also use ECS nanowires to achieve a ∆*T* better than 0.1 K in the human body temperature range (see SI Fig. S13 in Section IX).

The range of sensible temperature is another important attribute for a given PLT material or platform. In recent years, the extension of the sensing temperature range has received great attention from cryogenic temperatures to room temperature or even higher temperatures in a single material and device to meet the needs of different application scenarios. Figure 3e compares the temperature range with other approaches using various wavelength bands. The temperature range from 4 to 500 K of this paper is comparable with other approaches in the UV–VIS band and our temperature range are the widest in IR bands. At present, NIR-IIB wavelength bands are a fast-developing and highly interesting band for PLT in bio-medical applications where combined tissue scattering and absorption lead to the best tissue penetration in mammals. Figure 3f presents a summary comparison of key performance attributes between our approach and some of the best available approaches in terms of the range of sensible temperatures, sensitivity, and temperature resolvability (1/∆*T*). Notice that our results and ref. ^71^ are for NIR-IIB wavelengths, while others from the literature are for visible or shorter-wavelength IR. We see clearly that our present approach has obvious overall advantages in those key aspects. The only exception is the sensing temperature range. Some jobs use shorter wavelengths to make this parameter larger than ours, as shown in refs. ^51,72^.

**Fig. 4:**
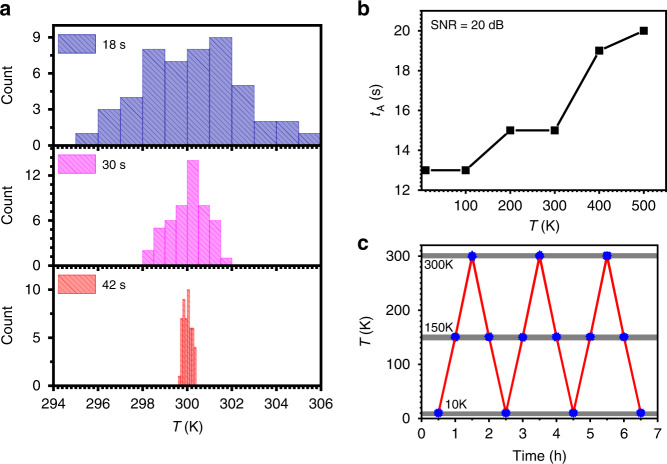
Acquisition time, resolution/SNR, and the measurement repeatability of a single ECS NW thermometer. **a** Measurement effect of target temperature 300 K with different acquisition time, **b** necessary time needed to reach SNR of 20 dB at different temperatures, and **c** repeatability test for three cycles with long working hours of our ECS NW thermometer

Figure 4 shows some key aspects of measurement errors and measurement repeatability. Figure 4a shows histograms corresponding to three different data acquisition times when the target temperature is 300 K. It is worth noting that the acquisition time *t*_A_ is inversely proportional to temperature resolution. As the measurement time increases, the measurement accuracy increases, as the standard deviation decreases from 2.23 to 0.85 to 0.18 K. This is because increasing *t*_A_ can improve the SNR of PLIR signal, resulting in better ∆T, as shown in Fig. S10d. Therefore, it may be necessary to decide which of *t*_A_ and ∆*T* is the priority in a given sensing scenario. Figure 4b shows the required data acquisition time to achieve a given SNR (defined by Eq. (S3)) of 20 dB vs. measurement temperature. As temperature increases, PL intensity decreases, requiring a longer measurement time to achieve the predetermined SNR. Repeatability is a vital parameter for a thermometer. One of the concerns with many nanomaterials is their stability under certain operating conditions. Figure 4c shows temperature measurements over three periods for 6 h when we cycled the temperature of the ECS nanowire from 10, 150 to 300 K three times. Every blue point is made up of 30 experiment data with an error bar of <1%. The result is robust with over 99.9% repeatability during different cycles. A more comprehensive list of performance comparisons is presented in Tables S3 and S4 in SI Section X, where we can acquire the numerical values of all key attributes for this work and significant literature. Furthermore, the effect of OH− ions and CO_2_ are considered because normally the traditional chlorides of rare-earth complexes such as ErCl_3_ are sensitive to these ions. Interestingly there is basically no change in the PL spectrum of a single ECS nanowire before the next year and one year after the storage condition without inert gas protection at room temperature, which is presented in Fig. S14 in SI section XI. Therefore, we believe that the effects of OH ions and CO_2_ absorption effects on the PL of ECS nanowires can be neglected.

Finally, we would like to address possible issues with polarization dependence of emission or absorption of our nanowire materials. Totally, the effective overall absorption would be affected, but the relative intensity ratio of various transitions would not be affected, since different transitions showed no dependence on polarization. The photoluminescence of the ^4^I_13/2_ levels was measured when either the polarization of the emission or the excitation laser was varied. The results are presented in Fig. S15. We find that the spectral shape of the emission does not change with the polarization of the excitation laser, but does show weak dependence on the collection polarizer. For a PLT with a non-polarized collection, the effects of polarized emission or absorption are very small, especially since we use intensity ratios. For a more detailed discussion on the polarization effect of the ECS nanowire photoluminescence, refer to Fig. S15 in SI Section XII.

## Discussion

To summarize, we have demonstrated a new strategy in this paper in photoluminescence thermometry that allows an automated optimization to choose the best intensity ratio as the sensing standard among several options, depending on the temperature range. Our procedure overcomes typical limitations of a single intensity ratio and does not rely on any prior knowledge of the temperature ranges of to-be-sensed objects. This new procedure can be used for various optimizations depending on the specific applications and associated requirements such as to have the best sensitivity, resolution, or precision.

Importantly, our approach was demonstrated using a single high-quality ECS nanowire, in contrast to the conventional approaches using a large collection of nanomaterials with non-uniform material properties. The single-nanowire approach represents a significant step towards size miniaturization and simplicity of PLT. Given the feasibility of recently demonstrated single-cell endoscopy using a nanowire^73^, we believe that there is a great potential for using similar single-nanowire single-cell thermometry for intra-cell probes of inner cell contents and activity. Raman-based temperature sensing has the advantage of not requiring a light emission process^74^. However, the spontaneous Raman signal, especially the anti-stokes signal is generally very weak and requires relatively high excitation power. Excessive excitation power will cause local temperature field changes. More importantly, the general cellular Raman signal is mostly based on the excitation in the visible band, and the background fluorescence will bring inevitable interference and poor SNR. The temperature resolution and accuracy of the Raman approach are typically worse than those of PLT. We have the ECS nanowires micro-manipulation experience on Si substrate^65^. A similar procedure may be useful for cells when we put nanowires on the tapered fiber, with glue fixed on the fiber and nanowire. A single nanowire can be injected into cells through 3-axis micromanipulators^73^. Considering the inorganic nature of ECS nanowires, we believe that they will have less cytotoxicity. The intracellular environment such as pH may have an effect on the signal intensity of individual PL peak intensity. But since we use the Stark sub-levels of the same energy level ^4^I_13/2_ for the ratio thermometry, the ratio mainly depends on the thermal distribution and should not be sensitive to other environmental variables. However, both the cytotoxicity and environmental effects are worth experimenting with proving, which is our present research interest.

The combination of the best PL material and our new strategy has led to PLT with an overall superior performance: the first sensitivity exceeding 100% K^−1^ (~138% K^−1^), the highest resolution of 0.01 K, the largest range of sensible temperatures of 4–500 K for the photoluminescence thermometry operating completely within 1500–1800 nm. Furthermore, if we combine a variety of optical characteristics (including intensity ratios between multiple sub-levels, even line widths, lifetimes, etc.) between the Stark sub-levels collected at different temperatures to form a high-dimensional parameter space, more sophisticated schemes such as neural network algorithms can be utilized for optimization. This could lead to a smarter and more accurate strategy for nano-thermometry. One potential issue with the PLT is the possible difference between extinction rates of the two wavelength bands used for the intensity ratio. This issue is severe if the two wavelength bands are far apart. In this sense, our present approach of using the Stark sublevels has some advantages because of the proximity of the Stark sublevels, as compared to the typical approaches where wavelength bands are further apart.

In addition, the best performance at cryogenic temperatures down to 4 K would have a significant impact in other high-tech areas such as quantum information and cryobiology. For example, cryogenics is crucial for macromolecular crystallography (MX) technology. Presently contact-based electric thermometers are mainstream for temperature determination in MX. The PLTs based on ensembles powders are introduced to this field recently for remote monitoring of temperature changes arouse from strong ionizing radiation^55^. For MX a remote cryogenic thermometer with a high spatial resolution is necessary to study the precise radiation damage of protein samples, but both technologies are unable to achieve high spatial resolution for the cryogenic temperature measurement. Our ECS single-nanowire PLT provides an alternative solution. Finally, we point out that this paper resolves several overall challenges of the current PLT approaches, and our approach is expected to play an important role both in conventional biomedical applications and in the emerging areas of cryobiology and quantum information technology.

## Materials and methods

### Materials

Si powder and ErCl_3_ powder (99.9%) were purchased from Sigma Aldrich, USA.

### Growth of ECS nanowires

ECS nanowires were grown using a vapor–liquid–solid (VLS) process assistant chemical vapor deposition (CVD) method and only small preparation details were changed compared with our previous technology. An Au film was coated on a silicon substrate by sputtering coating technology to make the VLS process available. Si powders in a small corundum crucible were placed upstream of the furnace of the CVD system, and 12 cm downstream we placed the ErCl_3_ powders in a small corundum crucible, another 7 cm away from the Au-coated silicon substrate lay. The 5% H_2_–Ar mixed gas was used as a carrier gas during material growth and the silica tube pressure was adjusted to 4 Torr with 50 sccm gas flow. The furnace of the CVD system was a single temperature zone tubular furnace and set at 1150 °C during nanowire growth.

### Characterization of ECS nanowires

The ECS nanowires were evaluated by scanning electron microscopy (SEM) and energy-dispersive X-ray spectroscopy (EDS), transmission electron microscopy (TEM), respectively. SEM and in-situ EDS measurements were performed utilizing a Philips XL-30 field-emission SEM equipped with an energy-dispersive X-ray detector. To acquire the elemental composition at each point on the sample, the substrate with the sample was fixed on an encoded *x*–*y* translation stage for locating the position accurately. The high-resolution TEM images were obtained by JEM-2100F TEM with an accelerating voltage of 200 kV. Samples were transferred to a 200-mesh copper grid using optical fiber probes under microscope observation. The samples on the substrate were placed onto the sample holder while a beam focusing on regions of nanowires was 0.5–1.0 mm.

### Photoluminescence (PL) spectrum and its temperature dependence experiments

The PL characteristics of ECS Nanowire were studied using a continuous-wave 974 nm laser as an excitation source with 70 µW output power. The nanowire was positioned on a Si substrate mounted on a 3-axis stage. The excitation spot size was about 2 µm focused by a ×100 objective lens. Sample emission was collected by the same ×100 objective lens and directed to a spectrometer and detected with a liquid nitrogen-cooled CCD detector. For the PL temperature calibration experiment from 4 to 500 K, the whole test system is the same as the PL experiment except that the sample is placed in a microscope cryostat (Oxford Instruments). Liquid helium was used to cool the samples to 4 K.

## Supplementary information

Support Information
