# Supplementary material for: Self-optimized single-nanowire photoluminescence thermometry
Source: Light Sci Appl. 2023 Feb 6;12:36. doi: 10.1038/s41377-023-01070-0 (PMC9899784; doi:10.1038/s41377-023-01070-0)
Supplement: Supplementary file 1 — Support Information [file 41377_2023_1070_MOESM1_ESM.docx]

**Supplementary Information for**

**Self-optimized single-nanowire photoluminescence thermometry**

*Zhang Liang*^1,2^*, Jinhua Wu*^1^*, Ying Cui*^1^*, Hao Sun*^1^*, and Cun-Zheng Ning*^1,2^***

^1^ Department of Electronic Engineering, Tsinghua University, 100084 Beijing, China.

^2^College of Integrated Circuits and Optoelectronic Chips, Shenzhen Technology University, 518118 Shenzhen, Guangdong, China.

^*^Communication author: Email: ningcunzheng@sztu.edu.cn

1. **Crystal structure, elemental analysis, and PL characteristics of ECS nanowires**
2. **Definition of characteristics**
3. **The theoretical foundation for the self-optimization strategy using Stark sub-levels**
4. **Experiment data and temperature measurement curve fitting for *R_1_* to *R_6_***
5. **The comparison of energy interval *ΔE_m_* and fitted slope *S_m_***
6. **The origin of deviation from the Boltzmann distribution for the lowest transitions**
7. **Determination of Threshold *R_th_* by *SNR***
8. **Self-optimization program for maximum sensitivity (by MatLab)**
9. **Performance improvement around body temperature using visible emission**
10. **Comprehensive performance comparison of various PLT approaches**
11. **The OH- ions and CO_2_ effect on the ECS nanowires.**
12. **Polarization characteristics of ECS nanowire photoluminescence**

**I.** **Crystal structure, elemental analysis, and spectrum characteristics of ECS nanowires**


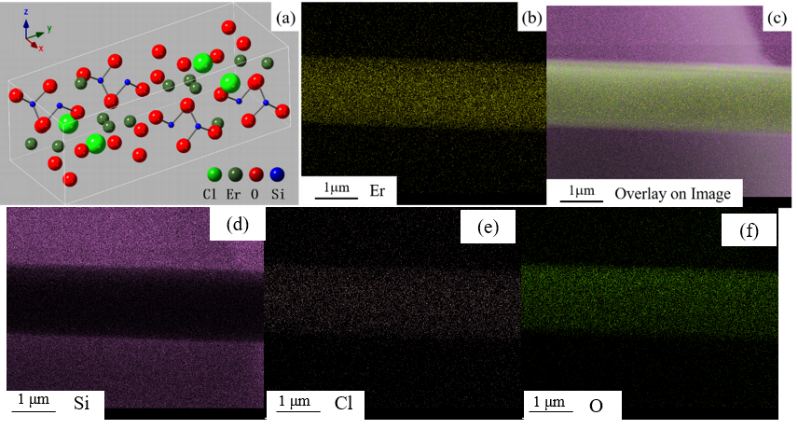


Figure S1. Material characteristics of ECS nanowire: a): ECS primitive cell; b) to f): EDS images of distributions of Er^3+^ ions (b), Si^4+^ ions (d), Cl^-^ ions (e), O^2-^ ions (f), and all the elements combined (c) (scale bar: 1 µm).


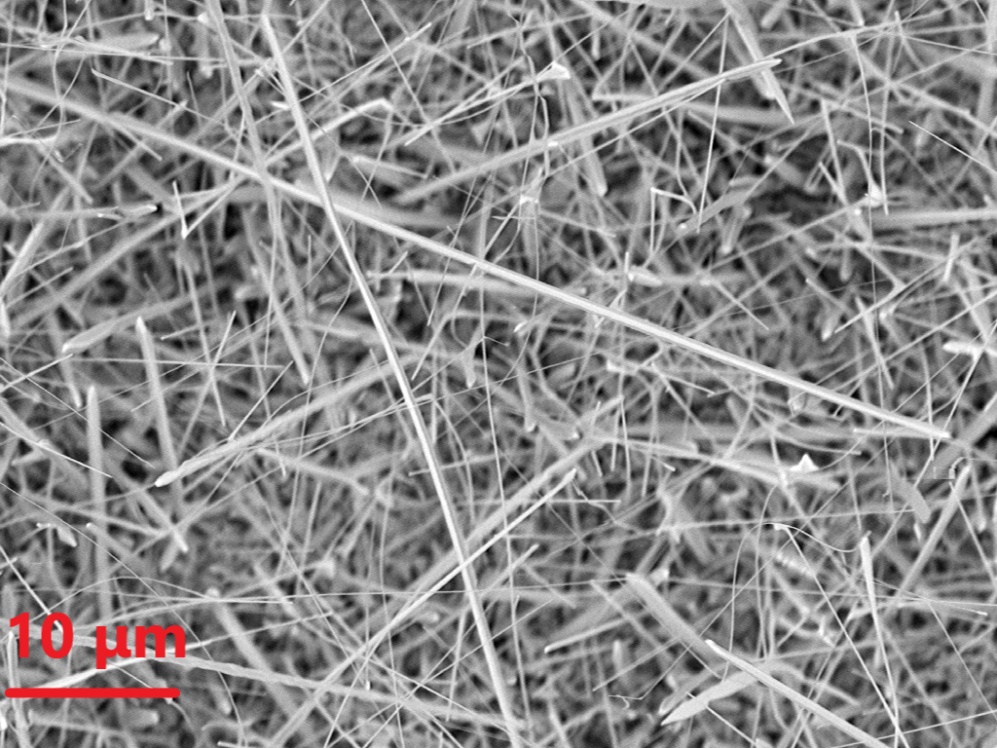


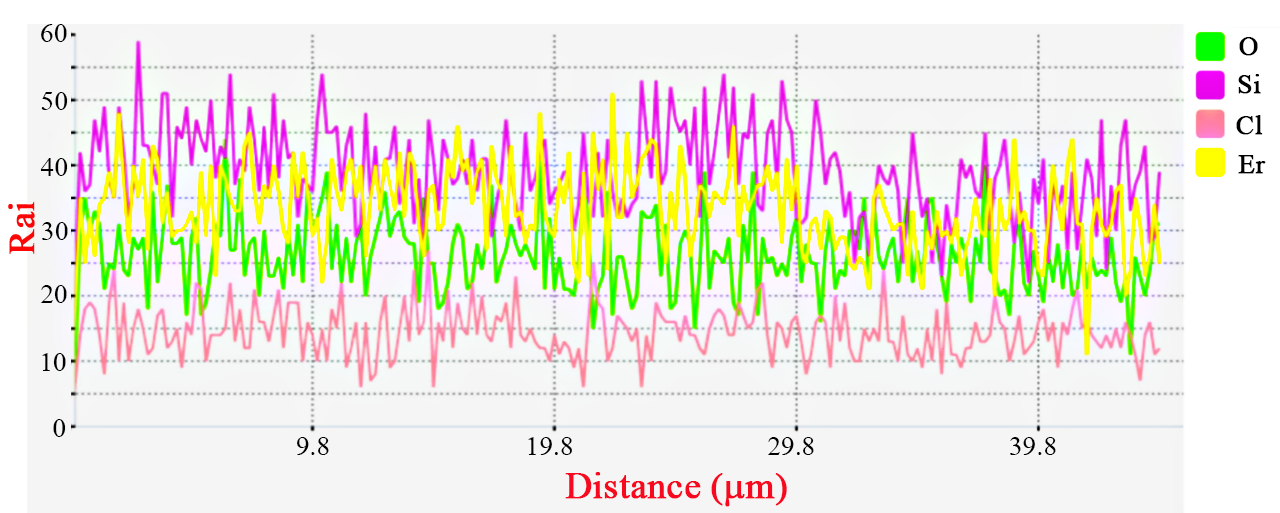


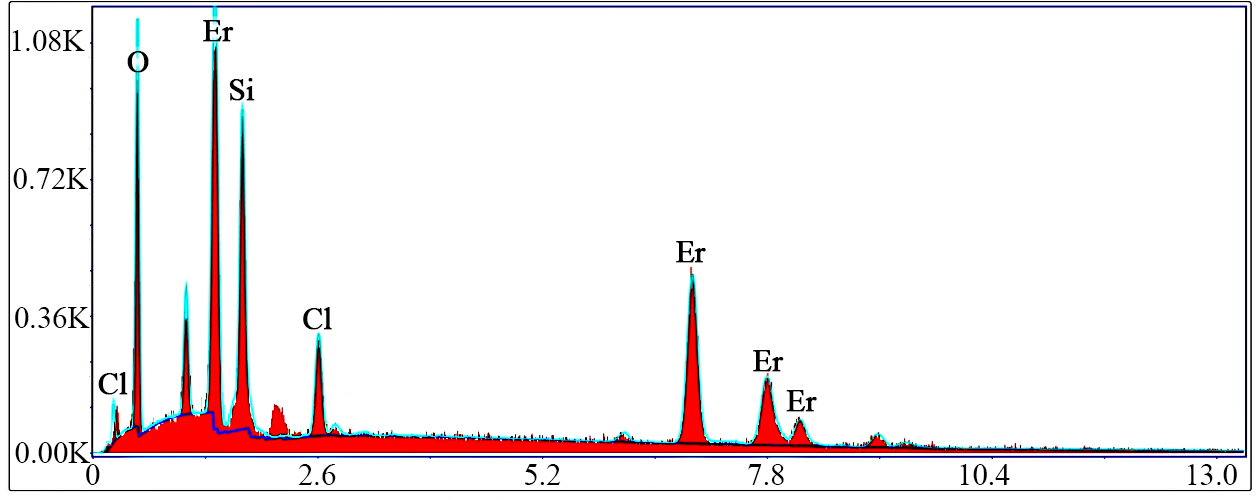


Figure S2. (a) The as grown ECS nanowires on Si substrate using the method described in the experiment part. (b) Line scanning energy dispersion spectrum (EDS) analysis of element ions of the ECS NW. (c) Dot EDS analysis of element ions of ECS NW.

Table. S1 Dot EDS element analysis of ECS NW.

| Element | Weight % | Atomic % | Error % |
| --- | --- | --- | --- |
| O | 18.22 | 50.24 | 10.40 |
| Si | 17.98 | 28.24 | 9.26 |
| Cl | 4.77 | 5.95 | 11.40 |
| Er | 59.03 | 15.57 | 5.36 |

**
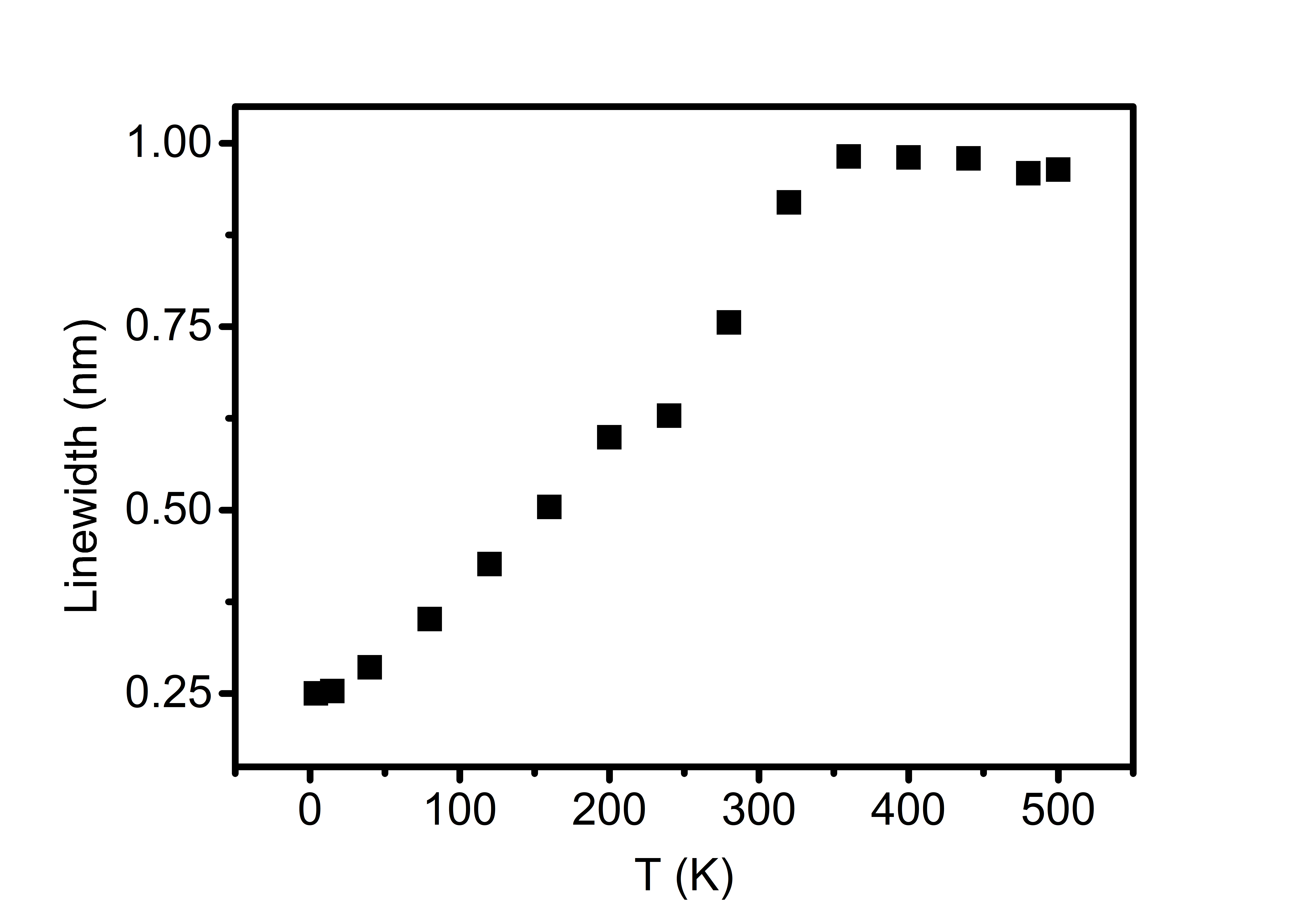
**

Figure S3. The linewidth evolution with temperature for the Stark sublevels.


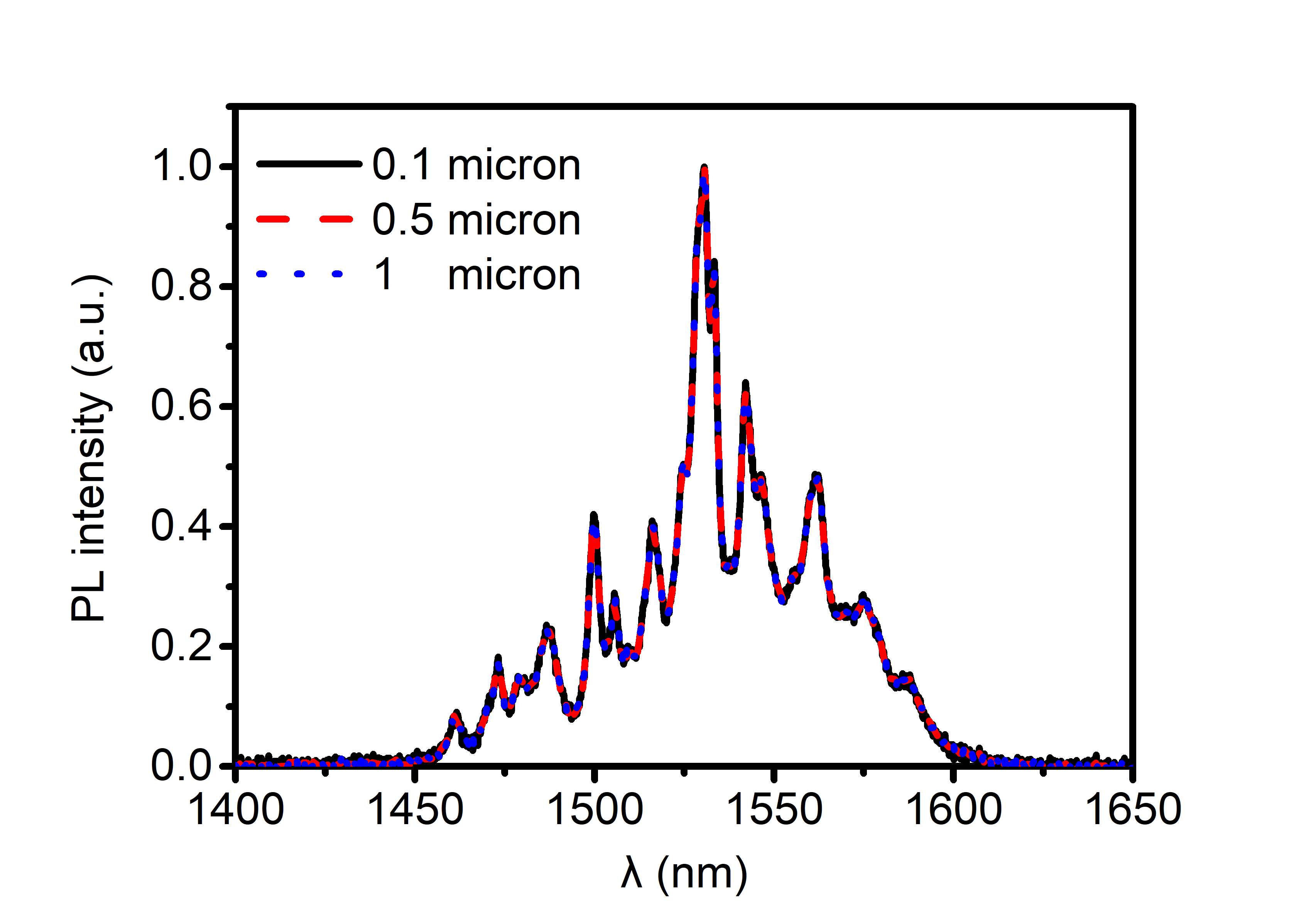


Figure S4. The normalized PL spectrum of the ECS NWs with diameter of 0.1, 0.5, and 1 micron at RT.

**II. Definition of characteristics.**

The standard deviation of the PLIR measurement is an indicator of a measurement’s precision and the dispersion of intensity ratios *R*. We name the standard deviation of *σ* and define as

$$\sigma=\sqrt{\frac{1}{N-1}\sum_{1}^{N} {(R_{i}-\overline{R})}^{2}} (S1)$$

where $\overline{R}$ represents the average of *N* repeated measurements for intensity ratios, *R_i_*

$$\overline{R}=\frac{1}{N}\sum_{1}^{N} R_{i} (S2)$$

Another important quantity is the signal to noise ratio (*SNR*) which can be defined as follows

$$SNR=10*\log\left( \frac{\bar{R}}{\sigma} \right) (S3)$$

The precision$\sigma_{T}$ of temperature measurement is defined as the standard deviation

$\sigma_{T}=\sqrt{\frac{1}{N-1}\sum_{1}^{N} {(T_{m}-\overline{T})}^{2}} (S4)$

Here *T_m_* stands for the temperature of the *m*-th measurement and $\overline{T}$ for mean temperature. So, the measured value of temperature is given by

$$T=T_{m}\pm\sigma_{T} (S5)$$

**Ⅲ. The theoretical foundation for the self-optimization strategy using Stark sub-levels**


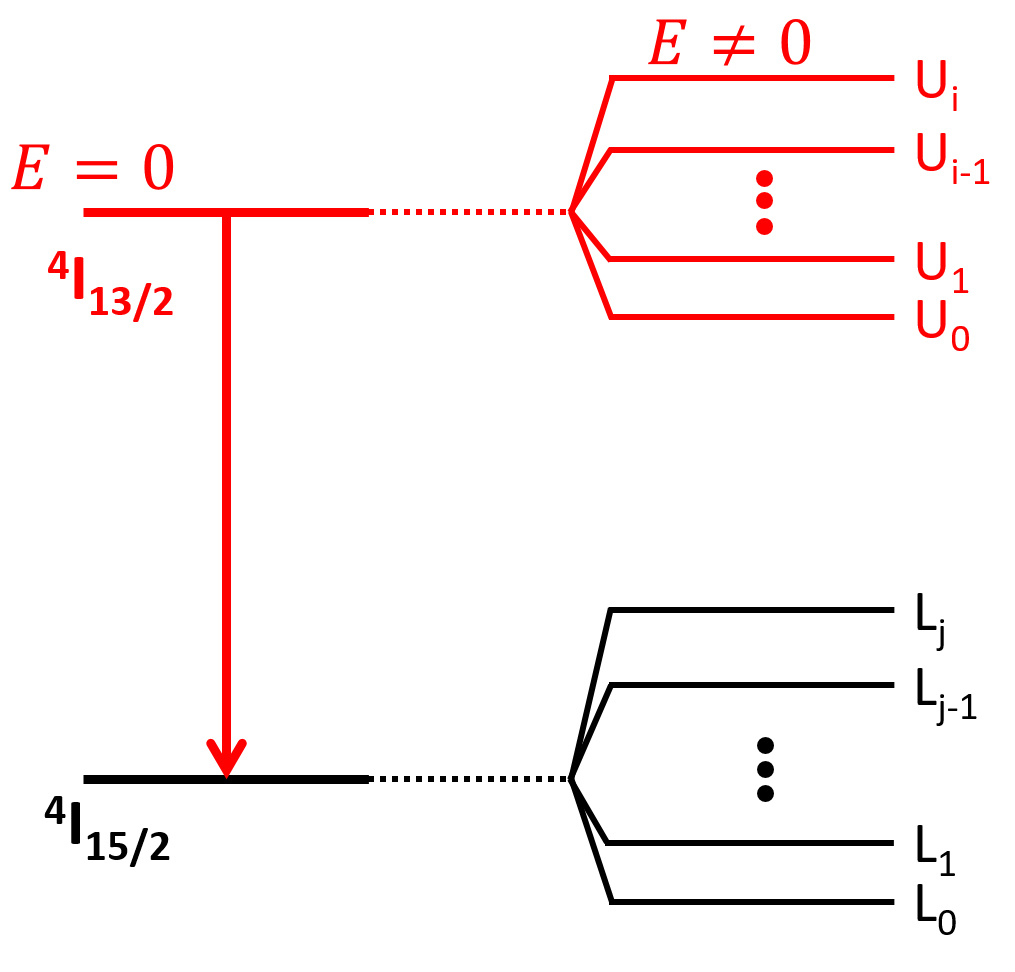


Figure S5. The schematic of the Stark sub-levels U_i_’s of ^4^I_13/2_ and L_i_’s of ^4^I_15/2_ levels of Er^3+^ ions in ECS lattice host with (*E*=0) and without (*E*=0) crystal field, *E*.

Here we use the ^4^I_13/2_ state and its Stark sublevels and the ^4^I_15/2_ state and its Stark sublevels for illustration, and the scheme described in the following should be valid for other energy levels and other ions. The discussion in the Section is the physics foundation of the self-optimization strategy presented in Fig. 2 of the main text. First the 980 nm pump laser populates ^4^I_11/2_ states directly. The rapid thermalization leads to a dynamic equilibrium, the Boltzmann, distribution among the ^4^I_13/2_ Stark sublevels. The transitions between ^4^I_13/2_ and ^4^I_15/2_ sub-levels generate multiple sub-peaks, reflecting the dynamic equilibrium Boltzmann distribution among the Stark sub-levels. This provides the physics foundation for PLIR thermometry based on the Stark sublevels. At the same time, the multi-peak feature provides an opportunity to construct multiple PLIRs, allowing us to choose the best one for thermal sensing based on optimization.

There are two methods to realize the thermometry which are named M1 and M2 respectively. For M1, we sum up all the PL intensity originating from a single ^4^I_13/2_ sub-level to various ^4^I_15/2_ levels and make ratios to get a response equation similar to traditional TC levels. The ratio of PL intensity of two sub-levels is like equation S6 below.

$$R\left( \frac{U_{m+k}-\sum_{n} L_{n}}{U_{m}-\sum_{n} L_{n}} \right)=\frac{I\left( U_{m+k}-L_{0} \right)+I\left( U_{m+k}-L_{1} \right)+I\left( U_{m+k}-L_{2} \right)+\cdot\cdot\cdot\cdot\cdot\cdot+I\left( U_{m+k}-L_{j} \right)}{I\left( U_{m}-L_{0} \right)+I\left( U_{m}-L_{1} \right)+I\left( U_{m}-L_{2} \right)+\cdot\cdot\cdot\cdot\cdot\cdot+I\left( U_{m}-L_{j} \right)} (S6)=\frac{\sum_{n} \left( hv\left( U_{m+k}-L_{n} \right)A\left( U_{m+k}-L_{n} \right) \right)N\left( U_{m+k} \right)}{\sum_{n} \left( hv\left( U_{m}-L_{n} \right)A\left( U_{m}-L_{n} \right) \right)N\left( U_{m} \right)}=\frac{N\left( U_{m+k} \right)}{N\left( U_{m} \right)}*\frac{\sum_{n} \left( hv\left( U_{m+k}-L_{n} \right)A\left( U_{m+k}-L_{n} \right) \right)}{\sum_{n} \left( hv\left( U_{m}-L_{n} \right)A\left( U_{m}-L_{n} \right) \right)}=C_{k}(M1)*exp(-\frac{E\left( U_{m+k} \right)-E(U_{m})}{k_{B}T})$$

Here, *A* stands for spontaneous emission rate of different transitions and independent of temperature, *h* is the Planck constant, *ν* represents emission frequency of different transitions, and *N* represents population density of energy levels.

In the cryogenic range near absolute zero, all electrons are basically at the lowest state *U_0_* of ^4^I_13/2_ levels. When temperature increases to *T_m_*, higher upper sub-levels would gradually be populated because of larger lattice thermal motion energy *k_B_T_m_.* Here we take 1/5 *k_B_T_m_* as a great judgment (1/5 is an empirical value). If the value is equal to the energy gap Δ*E_m_* (E(*U_m_*) – E(*U_0_*)), the energy is strong enough to populate *U_m_* through lattice phonon assistance. Thus, the lowest temperature limit *T_1_* depends on the energy gap Δ*E_1_* of *U_1_* and *U_0_* sub-levels. We can reach down to liquid helium temperature when the energy gap between sub-levels *U_0_* and *U_1_* is small like a few meV. Between temperature *T_m_* and *T_m+1_*, PLIR thermometry can be achieved by the emission intensity ratio of the sub-level *U_m_* and *U_0_* to ground states.


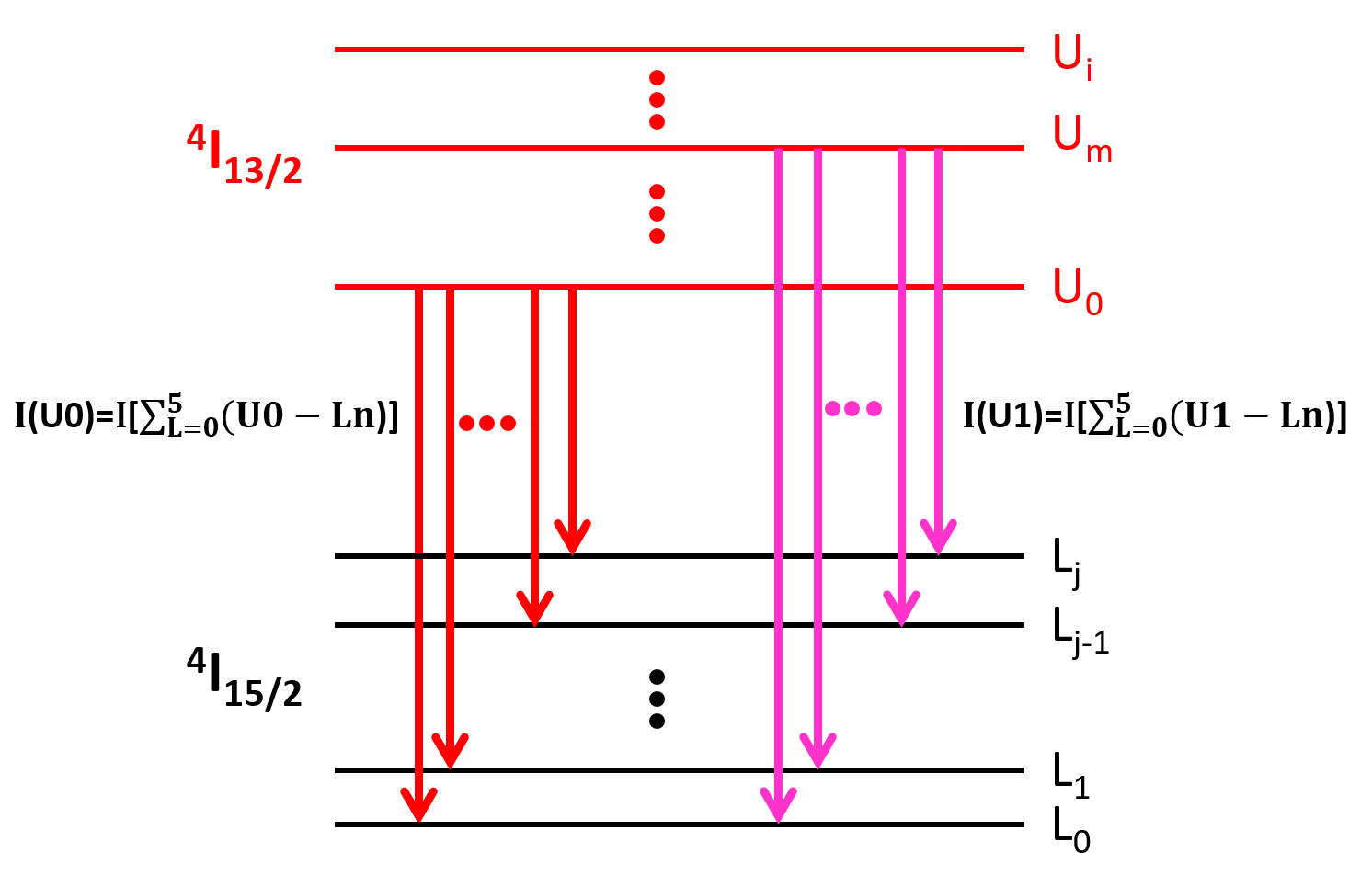


Figure S6. Various transitions from U_1_ and U_0_ sub-levels to ^4^I_15/2_ ground state. The total I(*U_1_*) and I(*U_0_*) can be used for the ratio for temperature measurement.

In another way, for M2, a single transition originated from an upper ^4^I_13/2_ sub-level to a lower ^4^I_15/2_ sub-level is used for PLIR thermometry as shown in equation S7.

$$R\left( \frac{U_{m+k}-L_{n}}{U_{m}-L_{n'}} \right)=\frac{hv\left( U_{m+k}-L_{n} \right)A\left( U_{m+k}-L_{n} \right)N\left( U_{m+k} \right)}{hv\left( U_{m}-L_{n'} \right)A\left( U_{m}-L_{n'} \right)N\left( U_{m} \right)}=\frac{N\left( U_{m+k} \right)}{N\left( U_{m} \right)}*\frac{hv\left( U_{m+k}-L_{n} \right)A\left( U_{m+k}-L_{n} \right)}{hv\left( U_{m}-L_{n^{'}} \right)A\left( U_{m}-L_{n^{'}} \right)} (S7)=C_{k}(M2)*exp(-\frac{E\left( U_{m+k} \right)-E(U_{m})}{k_{B}T})$$

Compared with M1, M2 brings us more convenience because we just have to make sure that two sub-peaks in the spectrum are originated from different upper sub-levels, but no need to pay special attention to exact lower sub-levels. This makes the practical operation of M2 much easier.

**Ⅳ. Experiment data and temperature measurement curve fitting for *R_1_* to *R_6_***

Temperature dependence of the 6 intensity ratios (*R_1_* to *R_6_*) obtained from experimental PL measurement was fitted assuming the strict Boltzmann distribution. The results are plotted in Fig. S7. The experimental points are shown as square dots. At each fixed temperature, we held for at least thirty minutes to assure that the thermal equilibrium was reached and the spectrum sample no less than twenty at each temperature point. The experimental data were fitted with strictly linear functions in the ln*R*-1/*k_B_T* coordinate system as shown in Fig. S7. The six response curves thus obtained are described in detail in Fig. 2b of the main text, and the parameter analysis and temperature measurement self-optimization scheme based on the fitted response curve is discussed in detail in Fig. 2 of the main text. At the same time, the strict linear function fitting can also explain that the method we use is based on the Boltzmann distribution principle. It is worth noting that we did not use the original value of *I_1_*/*I_0_* as the intensity ratio in the fitting of lowest transitions *R_1_*. Instead, a monotonic function relationship is established between (*I_1_*/*I_0_*-*C_M_*) as the intensity ratio and temperature. The physical origin of this constant is a deviation from Boltzmann distribution and is discussed in detail in SI section **ⅤI**.


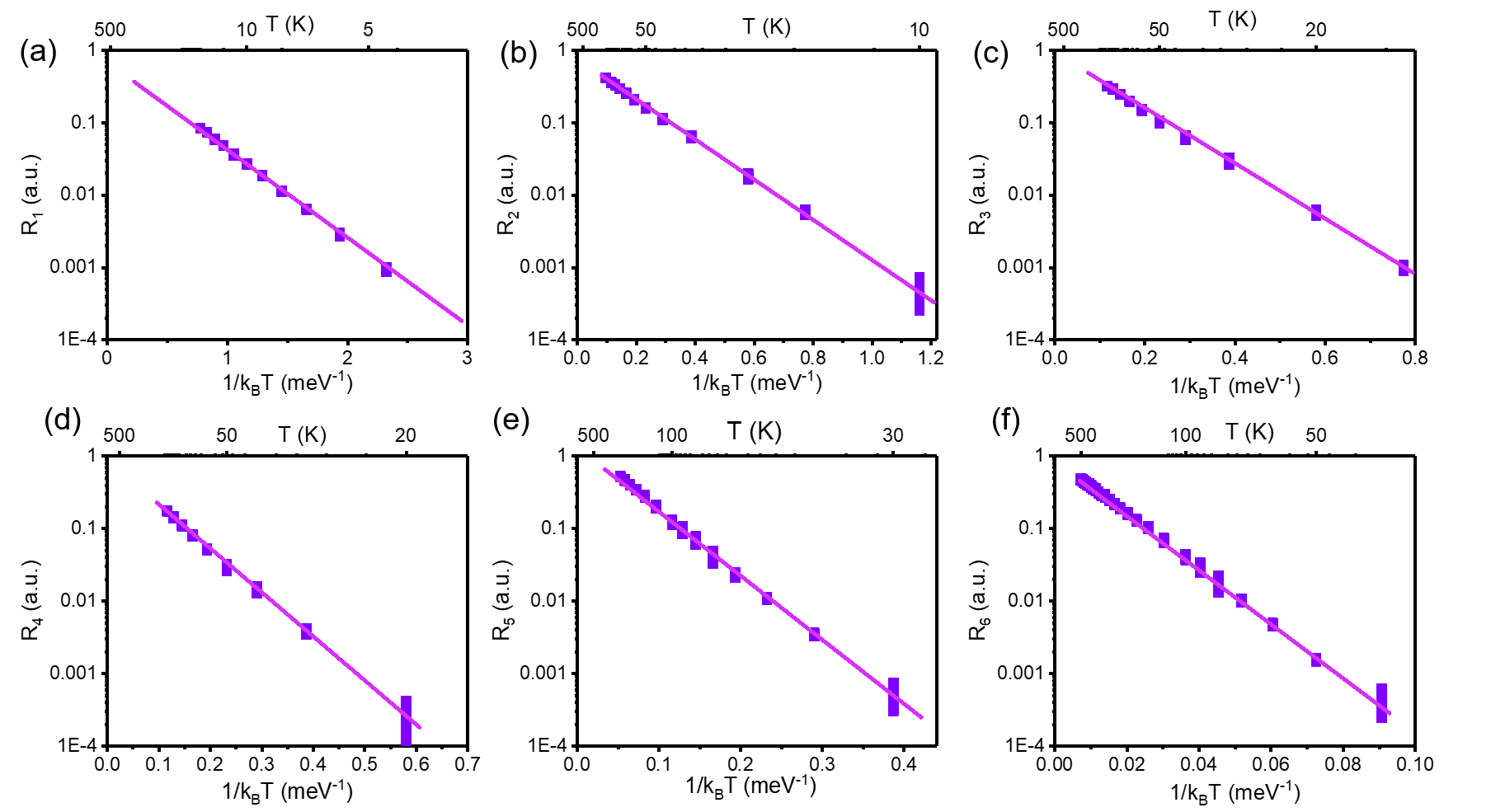


Figure S7. Experiment data (square dots) and fitting (solid lines) of intensity ratios (*R_1_-R_6_*) plotted on log-scale vs. the reciprocal thermal energy (bottom axis) with the corresponding temperature shown as the top axis.

**Ⅴ. The comparison of energy interval Δ*E_m_* and fitted slope *S_m_***

Table S2. Energy difference of chosen stark-sub-levels for PLIR thermometer.

|  | *R_1_* | *R_2_* | *R_3_* | *R_4_* | *R_5_* | *R_6_* | *R_7_* |
| --- | --- | --- | --- | --- | --- | --- | --- |
| *S_m_* (meV) | 1.8 | 4.8 | 9.3 | 18.0 | 24.3 | 32.8 | 4.3 |
| Δ*E_m_* (meV) | 1.4 | 4.8 | 9.6 | 18.2 | 25.3 | 33.2 | 8.0 |


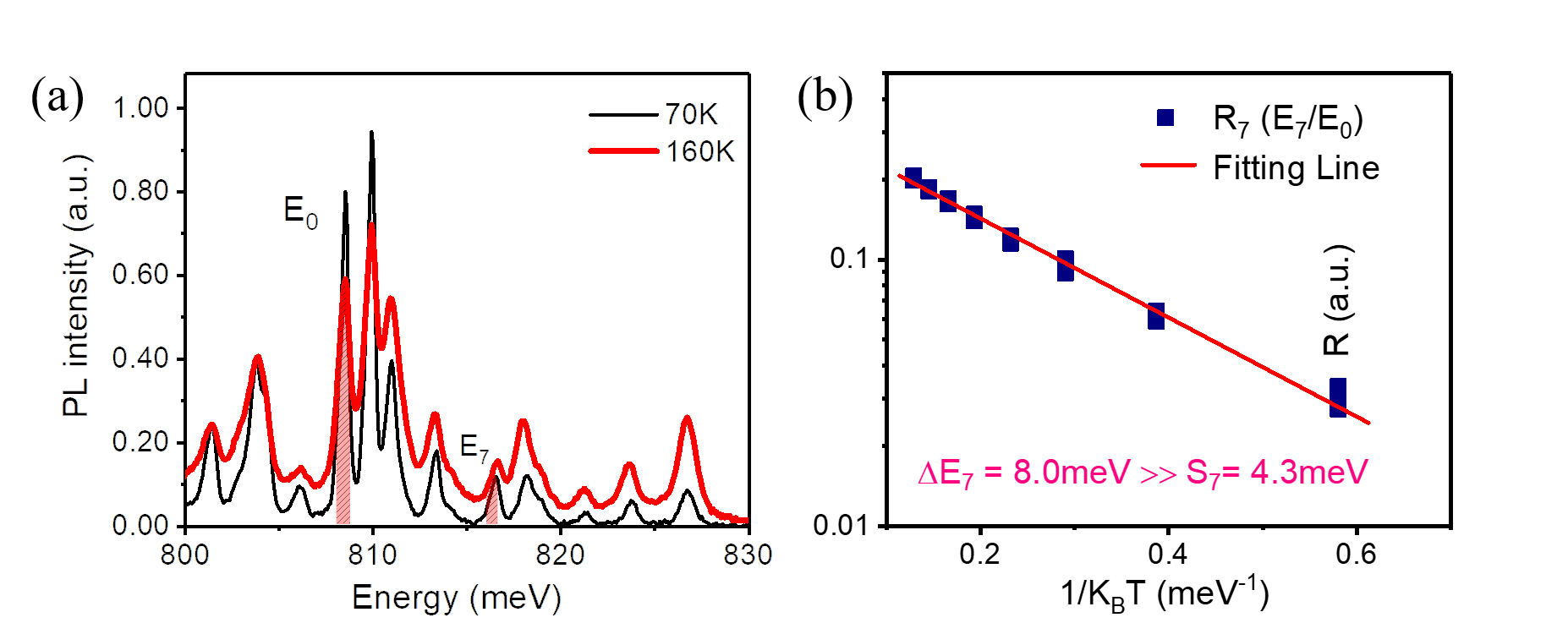


Figure S8. (a) The PL emission integration of *E_0_* and *E_7_*; (b) fitting slope of S_7_ between *E_7_* and *E_0_* as PLIR.

Δ*E_m_* represents the energy separation between *E_0_* and *E_m_*, obtained from the measured spectrum. *S_m_* represents the slope of the linear fitting in the ln*R_m_* -1/*k_B_T* plot. As we analyzed in the fourth step of the strategy for constructing PLIR, for the Boltzmann-type PLT, *S_m_* should have the same value as Δ*E_m_*. The six ratios obtained in our scheme all have the same *S_m_* and Δ*E_m_* values within the margins of error (see table S2). In addition, we analyzed an additional case *R_7_*. From Fig. S8 (a) and (b), we can get Δ*E_7_* and *S_7_*, respectively, and we find that the slope value of 4.3 meV obtained by fitting is almost half of the energy level interval of 8.0 meV obtained in the spectrum. This result is far beyond the error tolerance range. The deviation of *S_7_* and Δ*E_7_* is mainly originated from the fact that *S_7_* only reflects the energy difference between the upper energy levels of two transitions, while Δ*E_7_* reflects both the difference of upper and lower energy levels of the two transitions.

**Ⅵ. The origin of deviation from the Boltzmann distribution for the lowest transitions**

This deviation is mainly caused by the population distribution law of upper levels of *T_1_* and *T_0_* transitions. The correction factor *C_M_* in equation S12 is a sign of the deviation from the Boltzmann distribution. Here we try to analyze the origin of the deviation. Suppose that the upper and lower sub-levels of *T_1_* transitions are *U_1_* and *L_1_*, and by the same *U_0_* and *L_0_* for *T_0_* transitions. From the previous analysis, we know that the emission intensity ratio of *T_1_* and *T_0_* is decided by the population law of *U_1_* and *U_0_* sub-levels.

When *U_0_* and *U_1_* sub-levels become close or partially overlapped, the deviation from the Boltzmann will occur. As shown in Fig. S9, the population number *N_1_* of *U_1_* states can be divided into two parts of *N_1_'* and *∆N*. Similarly, population number *N_0_* of *E_0_* states can be divided into *N_0_'* and *∆N*. As we explained in equation S7, the PLIR for *U_1_*


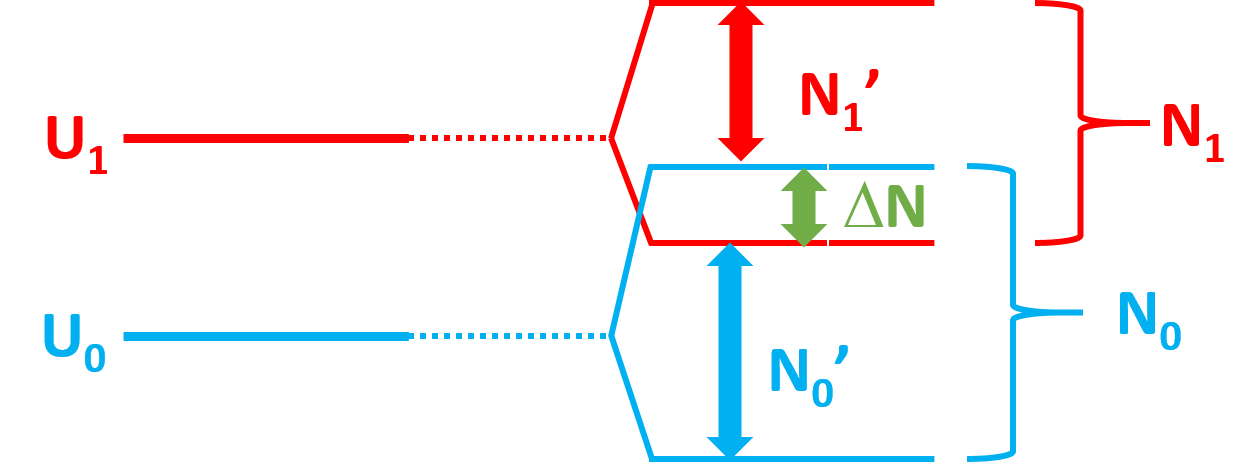


Figure S9. The possible overlap of *U_1_* and *U_0_* sub-levels via the Stark effect and the population distribution scheme.

and *U_0_* sub-levels can be written as equation S8 below

$$R_{1}=\frac{hv\left( U_{1}-L_{m} \right)A\left( U_{1}-L_{m} \right)N_{1}}{hv\left( U_{0}-L_{n} \right)A\left( U_{0}-L_{n} \right)N_{0}}=C_{1}*\frac{N_{1}}{N_{0}} (S8)$$

The ratio of *N_1_* and *N_0_* can be deduced as equation S9

$$\frac{N_{1}}{N_{0}}=\frac{N_{1}^{'}+\Delta N}{N_{0}^{'}+\Delta N}=\frac{{N_{1}^{'}}/{N_{0}^{'}}+{\Delta N}/{N_{0}^{'}}}{1+{\Delta N}/{N_{0}^{'}}} =\frac{N_{1}^{'}}{N_{0}^{'}}*\left( \frac{1}{1+{\Delta N}/{N_{0}^{'}}} \right)+\frac{1}{1+{N_{0}^{'}}/{\Delta N}} (S9)$$

The ratio ${\Delta N}/{N_{0}^{'}}$can be approximate to a constant, cause the two are originated from a single sub-level essentially.

$$\Delta N\left( T \right)\propto N_{0}^{'}\left( T \right) (S10)$$

So, we can rewrite equation S9 in the form of equation S11

$$\frac{N_{1}}{N_{0}}=\frac{N_{1}^{'}}{N_{0}^{'}}*\left( \frac{1}{1+{\Delta N}/{N_{0}^{'}}} \right)+\frac{1}{1+{N_{0}^{'}}/{\Delta N}}=a*\frac{N_{1}^{'}}{N_{0}^{'}}+b (S11)$$

The emission intensity ratio of equation S8 can be written as equation S12

$$R\left( \frac{U_{1}-L_{m}}{U_{0}-L_{n}} \right)=C_{1}*\frac{N_{1}}{N_{0}}=C_{1}*a*\frac{N_{1}^{'}}{N_{0}^{'}}+C_{1}*b=C_{1}*a*\frac{N_{1}^{'}}{N_{0}^{'}}+C_{M} (S12)$$

The $C_{1}$**b* is the origin of the deviation value *C_M_* of *R_1_*_._

**Ⅶ. Determination of Threshold *R_th_* by *SNR***

**
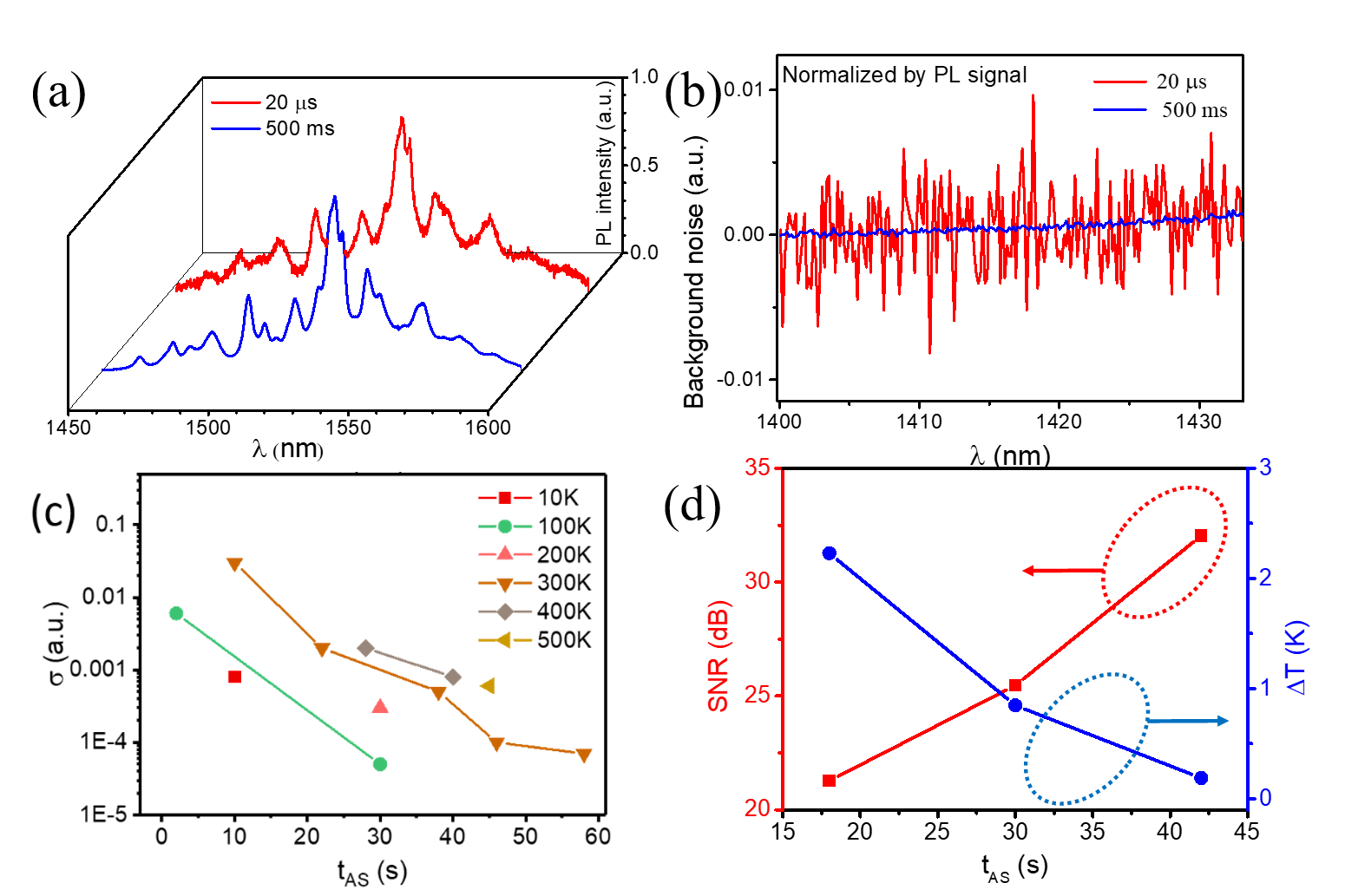
**

Figure S10. (a) PL spectrum at room temperature with different acquisition times with an ECS NW; (b) Comparison of normalized background noise under different acquisition times of an ECS NW; (c) σ vs. acquisition time at different temperatures; (d) *SNR* and resolution variation obtained using different acquisition times at room temperature.

Fig. S10 (a) depicts the normalized spectra obtained using different acquisition times of a single nanowire at the room temperature. It can be seen that as the acquisition time increases, the spectrum becomes smoother. But even in a very short time of 20 μs, we can still obtain a complete signal spectrum. Fig. S10 (b) shows the normalized background noise *(NBN*) with the maximum spectral signal defined by S8.

$$NBN = {I\left( \lambda\right)}/{I_{max}} (S13)$$

Here *I(λ)* stands for PL intensity of wavelengths with no characteristic emissions, and *I_max_* stands for the maximum PL intensity for transition ^4^I_13/2_ to ^4^I_15/2_. We can see that as the acquisition time increases, the background noise is accordingly reduced significantly. The reduction of background noise helps to reduce the standard deviation of the detected target ratio *R*. As shown in Fig. S10 (c), at a different temperature, the standard deviation of the fluorescence intensity ratio is significantly reduced with the increased acquisition time. The reduction of the standard deviation will then affect the *SNR* of the measured signal and the measurement resolution. As shown in Fig. S10 (d), the *SNR* can be increased from 20 to 32 at room temperature and the measurement resolution can be reduced from 9 K to less than 1 K. But the corresponding acquisition time has almost tripled. Therefore, there is a relationship between acquisition time and temperature measurement performance such as temperature resolution. One can either sacrifice resolution to do fast measurement or extend acquisition time to reach a better resolution, depending on different application scenarios.


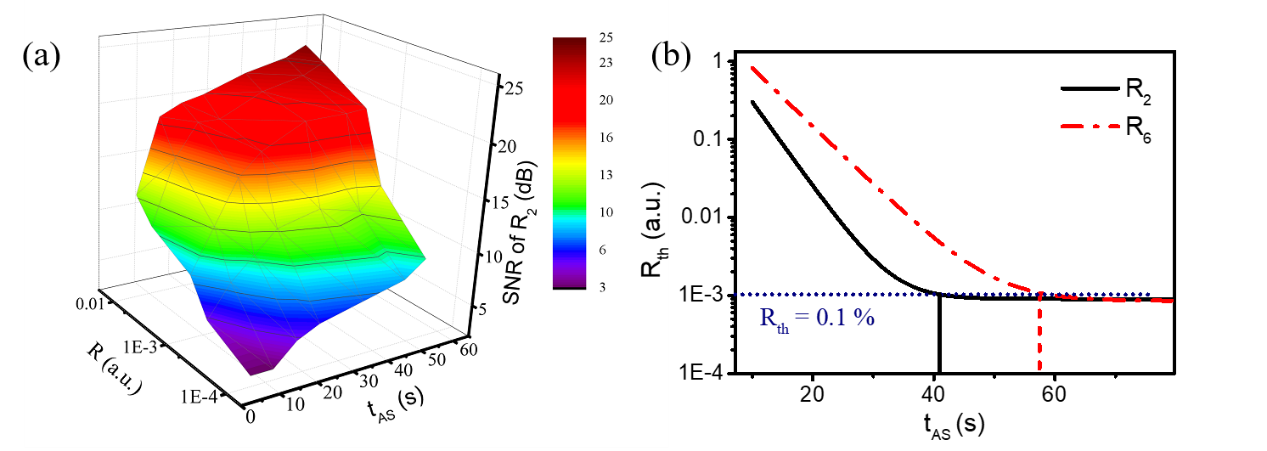


Figure S11. (a) *SNR* of *R_2_* vs. acquisition time and the *R* value; (b) *R_th_*-value and *t_AS_* relationship of *R_2_* and *R_6_* when *SNR* is set at 20 dB.

20 dB is generally regarded as a good *SNR* value [25][26]. Therefore, we use 20 dB as the threshold condition, as described in the self-optimization section of the main text. A minimum value of the intensity ratio, or the threshold *R_th_*, is required mainly to ensure that the signal value *R* has actual measurement significance within an acceptable acquisition time. Fig. S11 (a) shows the *SNR* results of multiple measurements of *R_2_* as a function of acquisition time and *R*-value. It can be seen that for significant ranges in the parameter space of (*R*, *t_AS_*), the *SNR* can be larger than 20 dB, indicating the validity of the measurements. Clearly the *SNR* = 20 dB will cut a line in the space of (*R*, *t_AS_*). This line defines a relation between *R_th_* and *t_AS_*. This is shown in Fig. S11 (b) for *R_2_* and *R_6_*, where we see that the threshold *R_th_* depends on the acquisition time. To simplify with a unified threshold in this paper, we choose a *R_th_* = 0.001 for all *R_i_* (i=1…6) (see Fig. 2b in the main text). As we see even for *R_6_*, a *SNR* of 20 dB can be acquired in 58 s at the threshold of *R_th_* = 0.001. By requiring a uniform *R_th_*, the lower temperature limit, *T_m_*, of each *R_i_* (i=1…6) can be determined as shown in Fig. 2b.

**Ⅷ. Self-optimization program for maximum sensitivity (by MatLab)**

A = readmatrix('xx.csv');

R1 = trapz(A(581:586,2))/trapz(A(596:606,2)); %initial judgement by R1%

if 0.4711 > R1;

syms x; % R_1_ for 4 to 9 K%

y = 0.446*exp(-21.9/x) == R1-0.432;

T = solve(y,x);

T = double (T);

display(T)

elseif 0.5455 > R1 >= 0.4711;

R2 = trapz(A(538:546,2))/trapz(A(596:606,2));

syms x; % R_2_ for 9 to 16 K%

y = 0.820*exp(-57.0/x) == R2;

T = solve(y,x);

T = double (T);

display(T)

elseif 0.6521 > R1 >= 0.5455;

R3 = trapz(A(479:489,2))/trapz(A(596:606,2));

syms x; % R_3_ for 16 to 31 K%

y = 0.947*exp(-107.7/x) == R3;

T = solve(y,x);

T = double (T);

display(T)

elseif 0.7031 > R1 >= 0.6521;

R4 = trapz(A(378:388,2))/trapz(A(596:606,2));

syms x; % R_4_ for 31 to44 K%

y = 0.82*exp(-205.0/x) == R4;

T = solve(y,x);

T = double (T);

display(T)

elseif 0.7315 >= R1 >= 0.7031;

R5 = trapz(A(297:307,2))/trapz(A(596:606,2));

syms x; % R_5_ for 44 to 55 K%

y = 0.92*exp(-294.7/x) == R5;

T = solve(y,x);

T = double (T);

display(T)

elseif R1 > 0.7315;

R6 = trapz(A(208:218,2))/trapz(A(596:606,2));

syms x; % R_6_ for 55 to 500 K %

y = 1.06*exp(-382/x) == R6;

T = solve(y,x);

T = double (T);

display(T)

else % beyond function range%

fprintf('%d. Beyond function! Error!!', n)

end

**Ⅸ. Performance improvement around body temperature using visible emission**

Figure S12. Visible PL emission processes of ECS NW under 980 nm excitation.

The PLT developed in this paper focuses mostly on the NIR-IIB BW. Therefore, we only needed to consider PLIRs for IR transitions from the sub-levels of ^4^I_13/2_. This has resulted in certain degradation of sensor performance in mammal body temperatures. For example, the sensitivity drops to below 1% K^-1^ and the temperature resolution is reduced to about 0.7 K. Although we can increase the temperature resolution by extending the measurement time and averaging, the sensing sensitivity will not be improved. We notice that the sensitivity is limited by fundamental physics in the IR wavelength ranges and not the result of our approach or our materials. For applications where sensitivity is essential, one can use the visible emission bands of ECS NWs. The high crystal quality of ECS NWs would still be important and advantageous in rapid acquisition time. Even under NIR (974 nm) excitation, we can observe a strong up-conversion visible PL signal. The upconversion mechanism is drawn in Fig. S12. Electrons are easy to be pumped to ^4^F_7/2_ states through an energy transfer process with ^4^I_11/2_ states. Lower states are populated along with a cross-relaxation process. Therefore, we can also use the classic ^2^H_11/2_ and ^4^S_3/2_ thermal coupling energy level pairs to measure temperature by ECS NW.


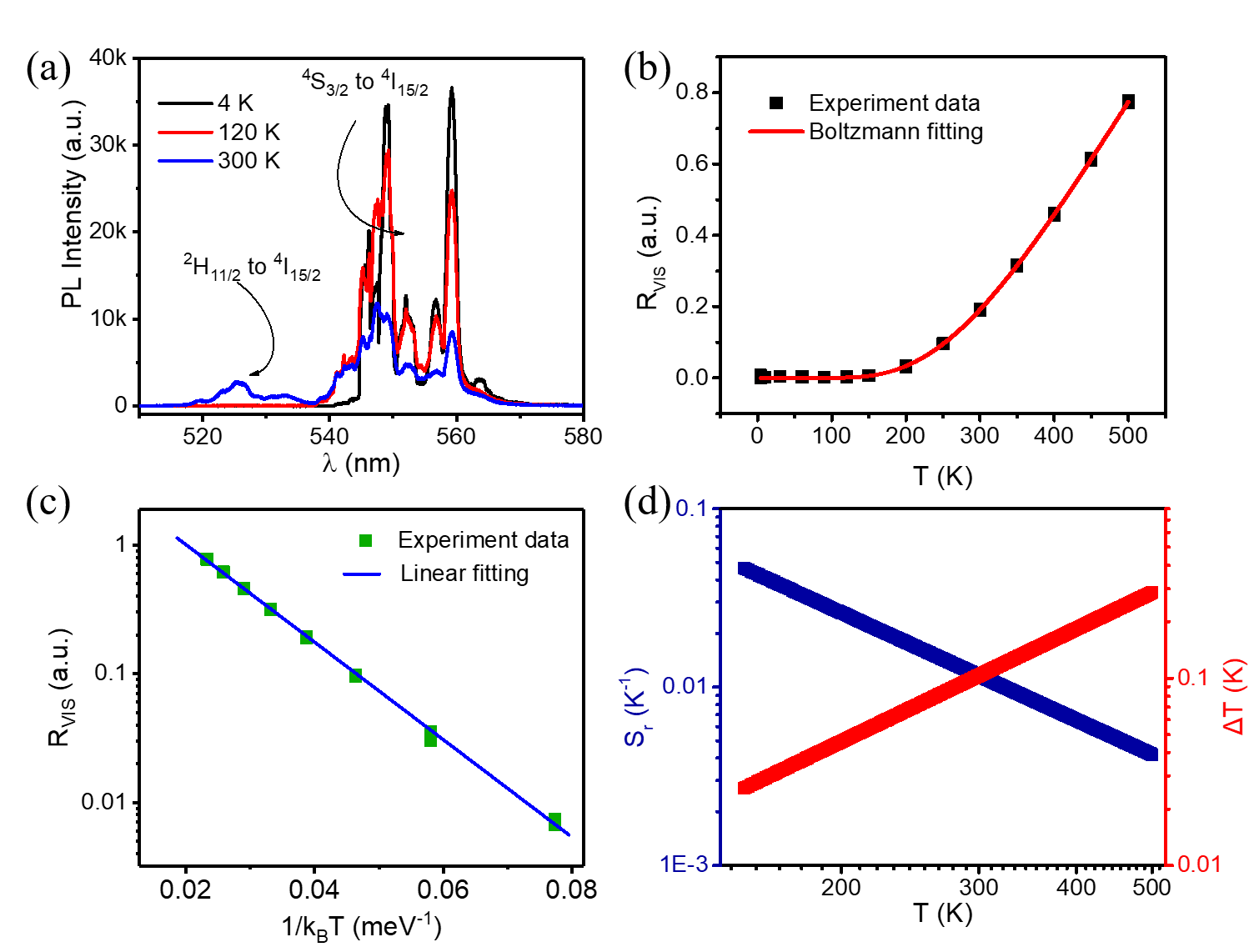


Figure S13. (a) The emission spectrum originated from classical TC levels of ^4^S_3/2_ and ^2^H_11/2_ of ECS NW; (b) PLIR experiment data and Boltzmann fitting vs. temperature; c) Same as in (b), but plotted against the reciprocal temperature; d) The relative sensitivity.

Fig. S13(a) shows the upconversion spectrum of ECS nanowire of traditional TCLs for ^2^H_11/2_ and ^4^S_3/2_ states. Fig. S13(b) shows the PLIR constructed from the two visible states, with black squares representing experiment data fitted with Boltzmann function (the red solid line). Fig. S13(c) is the experimental PLIR data in log coordinate as a function of 1/*k_B_T*, where the straight-line fitting reflects the strict Boltzmann distribution between ^2^H_11/2_ and ^4^S_3/2_ states. The sensitivity and resolution of this method are given in Fig. S13(d). Through this method, we can increase the body temperature sensitivity to above 1% K^-1^ while maintaining a temperature resolution of 0.1 K, as mentioned in the main text. This shows how our approach can be readily expanded into the visible spectrum to have higher performance around room temperature if a shorter spectrum is allowed for certain applications.

**Ⅹ. Comprehensive performance comparison of various PLT approaches**

In the main text, we have presented comparisons of the main performance attributes of our approach with those in literature in Fig. 3. In the following, we list the complete sets of these attributes to include actual numerical values.

Table S3. Comprehensive comparison of sensor performance (*I_ext_* and *I_em_* represent excitation and emission wavelength, respectively; symbol -, +, and & represent to, or, and, respectively; NG is not given in original papers).

| Mateiral | Range(K) | *I_ext_*(nm) | *I_em_*(nm) | *S_r_*(% K^-1^) | *ΔT*(K) | Ref |
| --- | --- | --- | --- | --- | --- | --- |
| ECS NW | 4-500 | 980 | 1530 | 138 | 0.01 | This work |
| Thermometry in NIR-IIB | | | | | | |
| LuVO_4_:Yb/Er | 300-500 | 980 | 1595&1660/  1595&1637 | 0.5 | NG | [1] |
| BaMoO_4_:Yb/  Er | 193-553 | 980 | 1504&1531/  1521%1531 | 0.095 | NG | [2] |
| (KLu(WO_4_)_2_:Tm)&(KLu(WO_4_)_2_:Tm/Ho)& (KLu(WO_4_)_2_:Tm/Yb) | 299-333 | 980 | 1710&1850/  1480&1711/  1711&1960/  1751&1805 | 0.6 | NG | [3] |
| LuVO_4_:Yb/Er@SiO_2_ | 303-353 | 915 | 1496&1527 | 0.185 | NG | [4] |
| KLu(WO_4_)_2_:Tm/Ho | 293-333 | 808 | 1450&1960/  1800/1960 | 0.9 | 0.55 | [5] |
| LiErF_4_@ LiYF_4_ | 293-318 | 793 | 1555-1573/  1609-1650 | 0.4 | NG | [6] |
| YAG:Yb/Er | 160-520 | 940 | 1450-1490/  1560-1590 | 1 | NG | [7] |
| Thermometry in other wavelengths | | | | | | |
| Nanodiamond | 110-330 | 532+637 | 595-620 | 10 | NG | [8] |
| Sc_2_O_3_:Eu^2+/3+^ | 77-267 | 254 | 403&612 | 3.06 | 0.08 | [9] |
| TTA-Nd-NPs | 283-323 | 635+808 | 540&1060 | 7.1 | 0.1 | [10] |
| NaYF_4_:Yb/Er | 298-330 | 980 | 525&545 | 1.15 | 0.1 | [11] |
| Sr$F_{2}$:Yb,Tm | 293-333 | 806 | 980&1010 | 1.62 | 1.7 | [12] |
| NaYF_4_:Er/Yb | 298-318 | 920 | 525&545 | NG | NG | [13] |
| SCFP:Eu^3+^ | 270-320 | 720 | 420&612 | NG | NG | [14] |
| LiLuF_4_:Nd^3+^ | 293-318 | 793 | 880&1050&1320 | 0.58 | 0.3 | [15] |
| Er-Yb@Yb-Tm LaF_3_ | 293-333 | 690 | 1000&1230&1550 | 5 | 0.3 | [16] |
| LiLuF_4_: $\text{Nd}^{3+}$ | 77-275 | 808 | 862&866 | 0.62 | 0.6 | [17] |
| Eu_0.95_Tb_0.05_-MOF | 40-300 | 322 | 543&615 | 0.17 | NG | [18] |
| SrGeO_4_:Pr^3+^ | 17-600 | 250 | 328&490&  608 | 9 | 0.1 | [19] |
| Tb_0.95_Eu_0.05_HL | 4-290 | 325 | 540&615 | 31 | 0.02 | [20] |
| $\alpha$-LiLuF_4:_ Tm^3+^@CaF_2_@NaYF_4_:Yb^3+^/  Er^3+^@CaF_2_ | 10-295 | 980 | 801&820/  521&538 | 0.67/  3.06 | NG | [21] |
| CaHfO_3_:Cr^3+^ | 40-150 | 450 | 700-800 | 2 | 0.045 | [22] |
| [(Tb_0.914_Eu_0.086_)_2_(PDA)_3_(H_2_O)]$\cdot$2 H_2_O | 10-325 | 377 | 540&615 | 5.96 | 0.02 | [23] |
| Na_2_K[(Lu_0.75_Yb_0.20_Er_0.05_)_3_Si_6_O_18_] | 12-450 | 903 | 930–1125/ 1425–1640 | 2.6 | 0.08 | [24] |

Table S4. Comparison of precision in NIR-IIB and visible (between ^2^H_11/2_ and ^4^S_3/2_ levels) using ECS NW.

| Temperature (K) | 10 | 100 | 150 | 300 | 350 | 400 | 500 |
| --- | --- | --- | --- | --- | --- | --- | --- |
| Precision by EST (K) | 0.05 | 0.27 | 0.46 | 1.07 | 1.9 | 2.7 | 5.8 |
| Precision by Visible (K) | × | × | 0.08 | 0.15 | 0.3 | 0.7 | 1.3 |
| *ΔT* by Visible (K) | × | × | 0.05 | 0.09 | 0.13 | 0.3 | 0.6 |

**Ⅺ. The OH^-^ ions and CO_2_ effect on the ECS nanowires.**

Under normal circumstances, rare earth chloride salt compounds are easily affected by ion molecules such as hydroxide OH^-^ ions and carbon dioxide in the air. For example, ErCl_3_ is particularly easy to absorb to form hexahydrate and saturated salt ErCl_3_.6H_2_O. PL would also be affected by these sensitizations and change a lot. Therefore, in order to verify the applicability of ECS nanowires, we compared the PL spectrum of a single ECS nanowire stored for one year in room temperature and air atmosphere. No change in intensity and line width was found as shown in Fig. S14. Therefore, we can infer that ECS nanowires are not sensitive to hydroxide ions and carbon dioxide molecules, and have strong practicality.


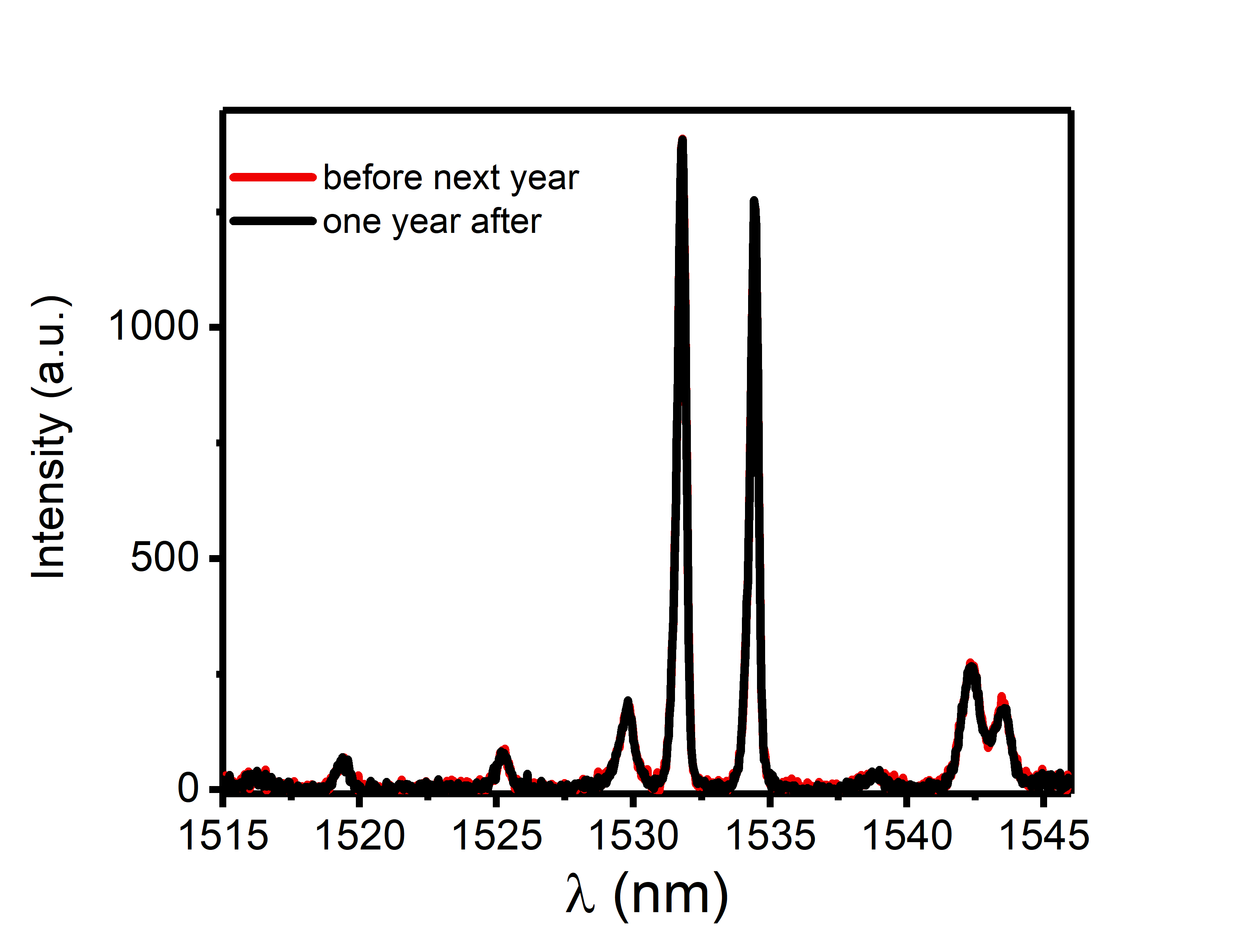


Fig S14. Comparison of PL spectra of single ECS nanowires before next year and one year after storage without inert gas protection at room temperature.

**Ⅻ. Polarization characteristics of ECS nanowire photoluminescence.**

Polarization resolved measurements were performed by either rotating the angle of the polarizer for the emission collection while fixing the polarization of the excitation laser or the other way around. The results are presented in Fig. S15. From Fig. S15a, b, c, and d, we find that the emission intensity of the nanowire has a significant polarization dependence. When the excitation polarization direction is parallel (Fig. S15a and S15b) or perpendicular (Fig. S15c and S15d) to the ECS nanowire, we find the amplitudes of Er^3+^ ions’ stark transitions do exhibit similar polarization dependence. We discussed the polarization degree which is defined as the following equation S14.

$$P=\frac{I_{max}-I_{min}}{I_{max}+I_{min}} (S14)$$

$I_{max}$ and $I_{min}$ denote the maximum and minimum values with different polarization directions, respectively. The polarization degrees for the emission are 25.8 % and 26.5 % when the excitation polarization is parallel and perpendicular to the b crystal axis (also the nanowire axis), respectively. As shown in Fig. S15e and S15f, the intensity ratio of the two-neighboring spectral (*R_1_*) peaks depends on the polarization much less sensitively. The polarization degrees of the emission for the intensity ratio are only 3.7 % and 3.4% for the two excitation directions. Fig. S15g and S15h respectively describe the excitation polarization characteristics of ECS nanowires. Fig. S15g depicts the normalized emission spectra between the excitation polarization directions from completely parallel to perpendicular to the nanowire. Fig. S15h shows the unpolarized collection of emission, the intensity ratio shows a much smaller degree of polarization (~ 0.8 %) as we rotate the polarization directions of excitation light. We should add that this small degree of polarization also includes possible experimental errors. In summary, even though the PL emission shows weak polarization dependence, the intensity ratio shows a maximum of 0.8 % error if unpolarized collection and polarized excitation are adopted. We have no good explanation at this point as to why the polarization is very weak. We suspect that the periodic environment of the Er atoms in the crystal makes the electronic transitions different from those of individual atoms in an Er doped material. This might be also related to the strong crystal field induced splitting of our ECS wires. But we would pursue the polarization properties more extensively in the future.

**
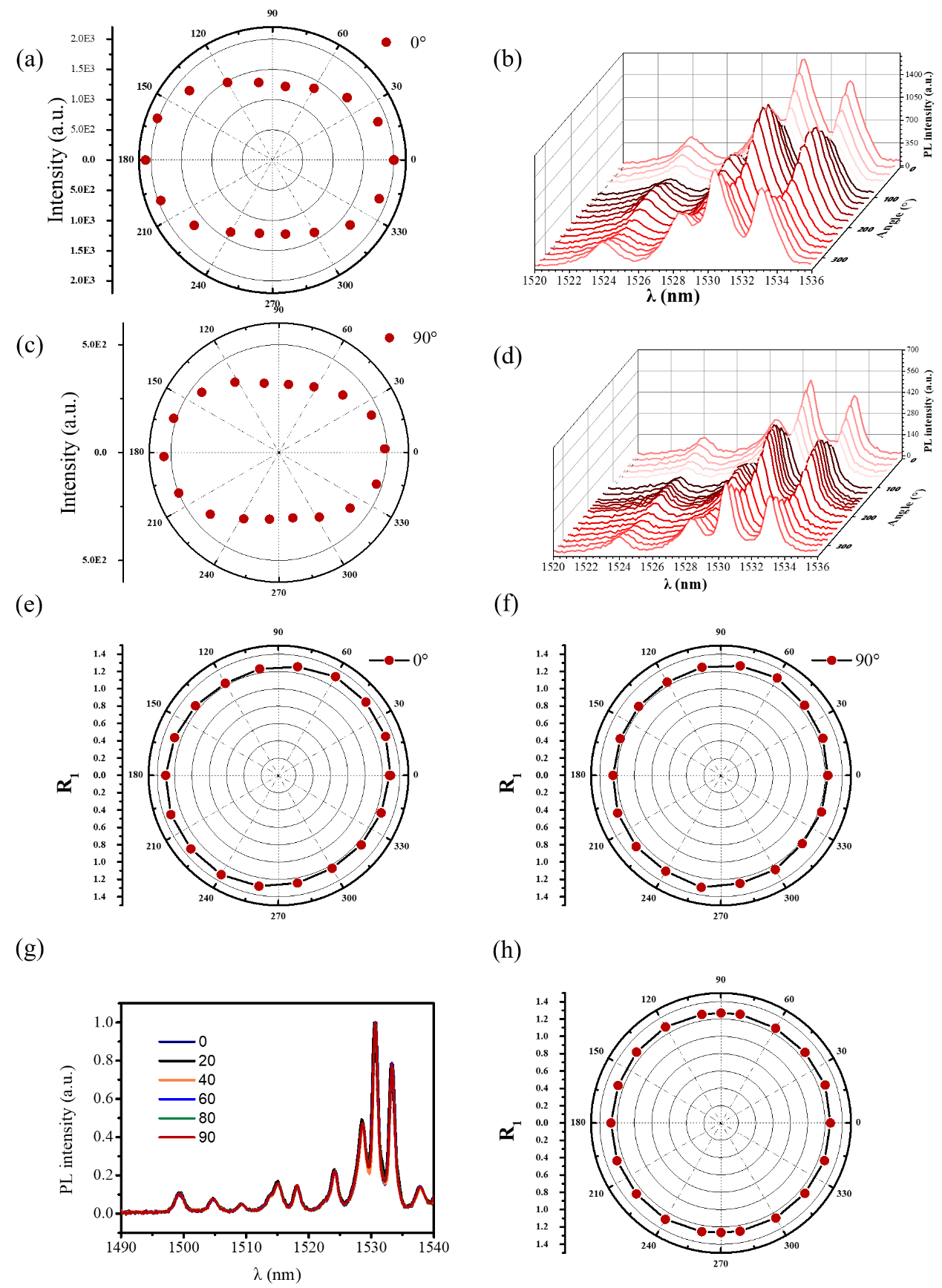
**

Figure S15. Polar plots of the emission intensity of the Stark sub-level E1 (as marked in Fig. g) as a function of the polarization angle with a step size of 20°, with the linear polarization of the excitation laser parallel (a) and perpendicular (c) to the nanowire; The corresponding spectra around 1530 nm are shown in Fig. (b) and (d), respectively. (e) and (f) show the polarization characteristics of the intensity ratio R1 with the collection polarization direction, with the linear polarization of the excitation laser parallel (e) and perpendicular (f) to the nanowire; (g) Normalized emission spectra of the single ECS nanowire recorded by rotating the polarized direction of excitation laser from 0 to 90°; (h) Polar plots of intensity ratio R_1_ as a function of polarization angle with the step size of 20° by rotating the polarized direction of excitation laser from 0 to 360°.

**References**

1. Ma, Y., *et al*. Upconversion properties and temperature sensing behaviors in visible and near-infrared region based on fluorescence intensity ratio in LuVO_4_: Yb^3+^/Er^3+^. Journal of Alloys and Compounds 769: 325-331. (2018).
2. Lei, R., *et al*. Optical thermometry based on anomalous temperature-dependent 1.53 μm infrared luminescence of Er^3+^ in BaMoO_4_: Er^3+^/Yb^3+^ phosphor. Optical Materials 86: 278-285. (2018).
3. Savchuk, O. A., *et al*. Luminescent nanothermometry using short-wavelength infrared light. Journal of Alloys and Compounds 746: 710-719. (2018).
4. Xiang, G., *et al*. Dual-Mode Optical Thermometry Based on the Fluorescence Intensity Ratio Excited by a 915 nm Wavelength in LuVO_4_:Yb^3+^/Er^3+^@SiO_2_ Nanoparticles. Inorganic Chemistry 58(12): 8245-8252. (2019).
5. Nexha, A., *et al*. Short-wavelength infrared self-assessed photothermal agents based on Ho, Tm: KLu(WO_4_)_2_ nanocrystals operating in the third biological window (1.45–1.96 μm wavelength range). Journal of Materials Chemistry C 8(1): 180-191. (2020).
6. Hazra, C., *et al*. "Erbium Single-Band Nanothermometry in the Third Biological Imaging Window: Potential and Limitations." Advanced Optical Materials 8(23): 2001178. (2020).
7. Periša, J., *et al*. All near-infrared multiparametric luminescence thermometry using Er^3+^, Yb^3+^-doped YAG nanoparticles. RSC Advances 11(26): 15933-15942. (2021).
8. Tran, T. T. *et al.* Anti-Stokes excitation of solid-state quantum emitters for nanoscale thermometry. *Science Advances* **5**, eaav9180, doi:10.1126/sciadv.aav9180 (2019).
9. Pan, Y. *et al.* Inherently Eu^2+^/Eu^3+^ Codoped Sc_2_O_3_ Nanoparticles as High-Performance Nanothermometers. *Advanced Materials* **30**, 1705256, doi:[10.1002/adma.201705256](https://doi.org/10.1002/adma.201705256) (2018).
10. Xu, M. *et al.* Ratiometric nanothermometer in vivo based on triplet sensitized upconversion. *Nature Communications* **9**, 2698, doi:10.1038/s41467-018-05160-1 (2018).
11. Brites, C. D. S. *et al.* Instantaneous ballistic velocity of suspended Brownian nanocrystals measured by upconversion nanothermometry. *Nature Nanotechnology* **11**, 851-856, doi:10.1038/nnano.2016.111 (2016).
12. Cortelletti, P. *et al.* Tuning the sensitivity of lanthanide-activated NIR nanothermometers in the biological windows. *Nanoscale* **10**, 2568-2576, doi:10.1039/C7NR06141B (2018).
13. Vetrone, F. *et al.* Temperature Sensing Using Fluorescent Nanothermometers. *ACS Nano* **4**, 3254-3258, doi:10.1021/nn100244a (2010).
14. Wang, X. D. *et al.* Two-Photon Excitation Temperature Nanosensors Based on a Conjugated Fluorescent Polymer Doped with a Europium Probe. *Advanced Optical Materials* **4**, 1854-1859, doi:[10.1002/adom.201600601](https://doi.org/10.1002/adom.201600601) (2016).
15. Skripka, A. *et al*. Advancing neodymium single-band nanothermometry. *Nanoscale* **11**, 11322-11330, doi:10.1039/C9NR02801C (2019).
16. Ximendes, E. C. *et al.* In Vivo Subcutaneous Thermal Video Recording by Supersensitive Infrared Nanothermometers. *Advanced Functional Materials* **27**, 1702249, doi: [10.1002/adfm.201702249](https://doi.org/10.1002/adfm.201702249) (2017).
17. Huang, P. *et al.* Unraveling the Electronic Structures of Neodymium in LiLuF_4_ Nanocrystals for Ratiometric Temperature Sensing. *Advanced Science* **6**, 1802282, doi: [10.1002/advs.201802282](https://doi.org/10.1002/advs.201802282) (2019).
18. Zhao, S. N. *et al.* Lanthanide Ion Codoped Emitters for Tailoring Emission Trajectory and Temperature Sensing. *Advanced Functional Materials* **25**, 1463-1469, doi: [10.1002/adfm.201402061](https://doi.org/10.1002/adfm.201402061) (2015).
19. Brites, C. D. S. *et al.* Widening the Temperature Range of Luminescent Thermometers through the Intra- and Interconfigurational Transitions of Pr^3+^. *Advanced Optical Materials* **6**, 1701318, doi:[10.1002/adom.201701318](https://doi.org/10.1002/adom.201701318) (2018).
20. Liu, X. *et al.* Mixed-Lanthanoid Metal–Organic Framework for Ratiometric Cryogenic Temperature Sensing. *Inorganic Chemistry* **54**, 11323-11329, doi:10.1021/acs.inorgchem.5b01924 (2015).
21. Shang, Y., *et al*. Dual-Mode Upconversion Nanoprobe Enables Broad-Range Thermometry from Cryogenic to Room Temperature. ACS Applied Materials & Interfaces 11(45): 42455-42461. (2019).
22. Back, M., *et al*. Pushing the Limit of Boltzmann Distribution in Cr^3+^-Doped CaHfO_3_ for Cryogenic Thermometry. ACS Applied Materials & Interfaces 12(34): 38325-38332. (2020).
23. Wang, Z., *et al*. Lanthanide–Organic Framework Nanothermometers Prepared by Spray-Drying. Advanced Functional Materials 25(19): 2824-2830. (2015).
24. Ananias, D., *et al*. Near-Infrared Ratiometric Luminescent Thermometer Based on a New Lanthanide Silicate. Chemistry – A European Journal 24(46): 11926-11935. (2018).
25. Malik, R. *et al*. Optical signal to noise ratio improvement through unbalanced noise beating in phase-sensitive parametric amplifiers. Opt. Express 22, 10477-10486 (2014)
26. Verma, D. Huawei Technologies Co., Ltd. How to calculate OSNR value of a WDM link (2012). at <https://support.huawei.com/enterprise/en/knowledge/KB0000592366>
